# Plume Dynamics Structure the Spatiotemporal Activity of Mitral/Tufted Cell Networks in the Mouse Olfactory Bulb

**DOI:** 10.3389/fncel.2021.633757

**Published:** 2021-04-30

**Authors:** Suzanne M. Lewis, Lai Xu, Nicola Rigolli, Mohammad F. Tariq, Lucas M. Suarez, Merav Stern, Agnese Seminara, David H. Gire

**Affiliations:** ^1^Department of Psychology, University of Washington, Seattle, WA, United States; ^2^Dipartimento di Fisica, Istituto Nazionale Fisica Nucleare (INFN) Genova, Universitá di Genova, Genova, Italy; ^3^CNRS, Institut de Physique de Nice, Université Côte d'Azur, Nice, France; ^4^Graduate Program in Neuroscience, University of Washington, Seattle, WA, United States; ^5^Department of Applied Mathematics, University of Washington, Seattle, WA, United States

**Keywords:** olfaction, olfactory navigation, plume dynamics, sensory processing, natural sensing, population dynamics

## Abstract

Although mice locate resources using turbulent airborne odor plumes, the stochasticity and intermittency of fluctuating plumes create challenges for interpreting odor cues in natural environments. Population activity within the olfactory bulb (OB) is thought to process this complex spatial and temporal information, but how plume dynamics impact odor representation in this early stage of the mouse olfactory system is unknown. Limitations in odor detection technology have made it difficult to measure plume fluctuations while simultaneously recording from the mouse's brain. Thus, previous studies have measured OB activity following controlled odor pulses of varying profiles or frequencies, but this approach only captures a subset of features found within olfactory plumes. Adequately sampling this feature space is difficult given a lack of knowledge regarding which features the brain extracts during exposure to natural olfactory scenes. Here we measured OB responses to naturally fluctuating odor plumes using a miniature, adapted odor sensor combined with wide-field GCaMP6f signaling from the dendrites of mitral and tufted (MT) cells imaged in olfactory glomeruli of head-fixed mice. We precisely tracked plume dynamics and imaged glomerular responses to this fluctuating input, while varying flow conditions across a range of ethologically-relevant values. We found that a consistent portion of MT activity in glomeruli follows odor concentration dynamics, and the strongest responding glomeruli are the best at following fluctuations within odor plumes. Further, the reliability and average response magnitude of glomerular populations of MT cells are affected by the flow condition in which the animal samples the plume, with the fidelity of plume following by MT cells increasing in conditions of higher flow velocity where odor dynamics result in intermittent whiffs of stronger concentration. Thus, the flow environment in which an animal encounters an odor has a large-scale impact on the temporal representation of an odor plume in the OB. Additionally, across flow conditions odor dynamics are a major driver of activity in many glomerular networks. Taken together, these data demonstrate that plume dynamics structure olfactory representations in the first stage of odor processing in the mouse olfactory system.

## 1. Introduction

Mice are adept at localizing odor sources (Gire et al., 2016; Baker et al., 2018; Liu et al., 2019; Gumaste et al., 2020), but the spatiotemporal information in olfactory environments that aids this search behavior is largely unknown. Odors travel in plumes which pull odor away from its source in filaments that are broken and distorted as they travel in air, creating complex odor environments. From the perspective of an olfactory searcher, these intermittent filaments create stochastic odor encounters, or whiffs, such that odor concentration dynamics fluctuate rapidly from moment to moment. Features of these complex plume dynamics contain information regarding odor source location (Murlis et al., 2000; Celani et al., 2014). For example, as a searcher encounters odors, the frequency, strength, and timing of encounters provide complex cues about an odor source (Atema, 1996; Vergassola et al., 2007; Ache et al., 2016; Michaelis et al., 2020).

A simple strategy, such as averaging odor concentration across whiffs could eliminate the complexity of an odor plume, allowing an animal to simply follow an increasing odor concentration gradient to the odor source. However, an animal dependent on this search strategy would operate at a timescale far slower than that observed in mice engaged in olfactory-guided search (Gumaste et al., 2020). This suggests that rodents most likely extract information from the complex spatiotemporal dynamics of olfactory environments to support their efficient odor-guided search behavior.

The extraction of information from fluctuating odor plumes will necessarily be impacted by the physics of odor transport. Factors such as wind speed and the Reynolds number of the plume could impact early olfactory processing in mammals. Precise olfactometers have been used to model certain features found in natural odor environments, such as fluctuating and intermittent odor concentration dynamics. Although this work provides important insights, olfactometers do not capture the full complexity of the odor environment. One problem is a lack of knowledge regarding which features of the plume are relevant to olfactory search, constraining which features olfactometers have been used to mimic. In addition, olfactometers create artificial plumes that decouple odor concentration from features present in olfactory environments. This omits correlations between concentration fluctuations and the surrounding air flow as well as small scale details of odor transport like diffusion. Decoupling these factors creates challenges for interpretation because it implicitly disrupts processing moderated by these features, such as the impact of wind speed on the vibrissal system (Yu et al., 2016) or feedback regarding bilateral nasal sampling (Markopoulos et al., 2012; Esquivelzeta Rabell et al., 2017). Directly observing how MT activity is impacted by the plume dynamics of natural olfactory scenes will thus constrain hypotheses regarding which spatiotemporal features of natural odor stimuli are conveyed by the brain.

We studied the response of MT cells in the OB to odor concentration dynamics in awake mice as they processed natural olfactory scenes, i.e., odor plumes. We used wide-field calcium imaging to measure MT activity, allowing us to study MT activity contributing to OB output at the level of glomerular complexes on the dorsal surface of the OB. Simultaneous recordings of the OB and plume dynamics show glomerular population activity follows fluctuations of odor concentration during plume encounters. The reliability and following behavior of glomerular responses were moderated by wind speed and the resulting changes in plume structure. The fidelity of odor concentration tracking increased when concentration dynamics were skewed, creating intermittent odor encounters across the plume presentation. In addition, as the strength and reliability of odor-evoked activity in MT cells increased, this activity more accurately followed plume dynamics. Together, these data demonstrate for the first time that the rapid fluctuations present in natural olfactory scenes significantly structure the activity of glomerular MT cell populations in the mouse OB.

## 2. Materials and Methods

### 2.1. Olfactory Stimuli

**Figure 1:**
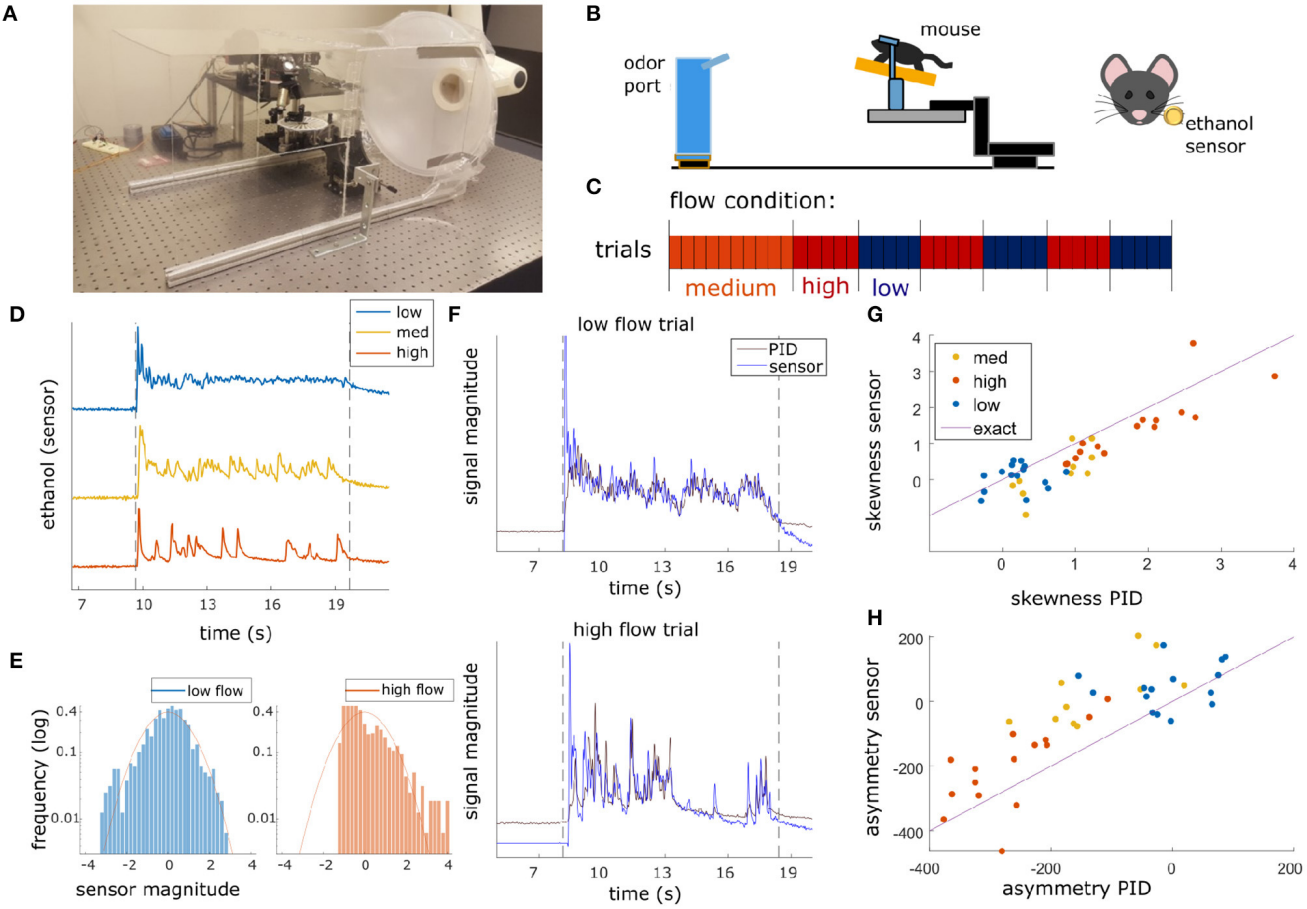
Plume presentations and head-fix setup for *in-vivo* recording experiments. **(A)** All experiments conducted in a 40 × 40 × 80 cm wind tunnel for quick clearing of odor presentations. The odor port (not pictured) was located ~13 cms upwind of the animal's nose. **(B)** (Left) Graphic detailing experimental setup. (Right) Ethanol odor concentration measured using a modified, commercially available ethanol sensor placed ~4 mm from the outer edge of the mouse's nostril. **(C)** Diagram depicting flow conditions (high, medium, or low) of the 40 trials within a single session. **(D)** Example odor traces are depicted for each flow condition. **(E)** Histograms of the odor concentration magnitude sampled across two examples trials show a change in skewness between low flow (left, blue) and high flow (right, red), with skewness increasing with increased airflow during plume presentations. **(F)** Comparisons between the deconvolved sensor signal and a PID signal during a set of paired recordings show odor concentration dynamics of the deconvolution can recover dynamics observed in the PID recordings (*r* = 0.61, *p* < 0.001). An example from low flow (top) and high flow (bottom) are shown. **(G,H)** Skewness **(G)** and asymmetry **(H)** of the deconvolved ethanol signal vs. the PID traces for each trial. All points lie close to the bisector (Purple line, labeled “exact”) showing that the deconvolution preserves measures of skewness and asymmetry consistent to the PID trace. High flow trials (orange) are separable from low flow trials (blue) and are substantially different from 0 (the expectation for any symmetric distribution, e.g., a Gaussian).

Olfactory stimuli were released by an automated odor port within a 40 × 40 × 80 cm acrylic wind tunnel where airspeed was controlled by a vacuum at the rear of the wind tunnel, posterior to the animal's location (Figure 1A). Concentration dynamics of olfactory stimuli varied stochastically from trial to trial creating plumes with unique concentration dynamics on each trial. Adjusting the velocity of wind flow allowed for variation in the Reynolds number, resulting in characteristic changes of plume dynamics for different flow levels (Supplementary Figure 1). Reynolds numbers were calculated using the mean flow of each condition and the half height of the tunnel (20 cm) as the head-fix setup is placed on a stage such that it is elevated ~20 cm from the tunnel floor (Figure 1B). Low, medium, and high flow Reynolds numbers were 2,400 ± 1,000, 8,800 ± 400, and 9,800 ± 200, respectively (mean ± st. dev), hence the medium and high flow conditions are fully turbulent, while the low flow is in the chaotic mixing regime. Across all flow conditions, plume presentations are stochastic and plume dynamics do not correlate across time within trials (Supplementary Figure 2A) or across trials (Supplementary Figure 2B). Odor statistics confirm that high and medium conditions are fully turbulent (as seen from large skewness levels implying intermittency (Figures 1E–G) and spectra close to the expectation for turbulent flows, see Supplementary Figure 1D). The low flow condition is not yet fully turbulent [as seen from the low values of skewness and symmetric odor distribution (Figures 1E,G) and flatter spectrum (Supplementary Figure 1D)]. Absolute velocities of low, medium and high flow conditions were 0.40 ± 0.16, 1.31 ± 0.05, and 1.81 ± 0.17 fpm and velocity fluctuations were 14 ± 3, 6 ± 2, and 5.3 ± 1.5 fpm, respectively.

A single session consisted of forty trials of odor presentation. Odor ports were located upwind ~13 cm anterior to the animal's nose (Figure 1B), and each plume presentation had a duration of 10 s. Odor release began ~10 s into the trial. In order to avoid responses predicting the beginning of the plume, the exact time of odor release was randomized by adjusting the duration of the 5 s intertrial interval by a length of time (s) drawn randomly from a uniform distribution, *U*(−2, 2). Random clicking noise was used to control for the clicking sound of the port serving as a cue for plume onset. Starting 5 s prior to plume onset, a number was drawn from a uniform distribution, *U*(0, 1), for each camera frame, and if the number exceeded 0.95 a clicking sound was produced.

Ethanol concentration throughout each trial was measured by a modified, commercially available ethanol sensor placed within 3.5–4 mm from the mouse's right nostril (Figure 1B). For the experimental flow sessions, a benzaldehyde and ethanol odor solution (0.6% benzaldehyde, 85.7% 200 proof ethanol, and 13.7% distilled water) was used as the plume source for all trials. Odor solutions were stored in odor reservoirs (centrifuge tubes) with air-tight, customized tops. Tops had two openings connected to tubing. One tube was connected to a Clippard electric valve (part no. EV-2-12) to create airflow and the other was attached to a 3D printed odor port. For each plume presentation, the valve was opened to allow airflow into the tube such that odor vapors exited the odor reservoir and traveled through cylindrical tubing (1/16″ inner diameter) to the release point at the odor port. The odor port mounted the end of the tubing so that it was suspended at roughly nose height and located ~13 cm directly upwind of the animal's nose (Figure 1B).

One set of experiments consisted of an odor panel during which 3 odors were released across forty trials. For these sessions, wind speed was held constant and only odor changed. For all trials plumes were presented at high flow. For the first ten trials, ethanol was presented (solution for plume source 86.3% 200 proof ethanol and 13.7% distilled water), for trials 11–25 a benzaldehyde-ethanol mixture was presented (solution same as flow experiments), and for trials 26–40 a isoamyl acetate- ethanol mixture was presented (solution for plume source consisted of 0.6% isoamyl acetate, 85.7% 200 proof ethanol, and 13.7% distilled water). Solutions were stored in three separate reservoirs, each with its own separate value and tubing. To change between odors during a session, the tubing running into the odor port was switched out manually during the appropriate intertrial intervals dividing odor conditions.

### 2.2. Implantation of Cranial Window

Implantation of the cranial window was adapted from methodology detailed in Batista-Brito et al. (2017). Mice (*n* = 3 flow panel, *n* = 2 odor panel) were anesthetized with isoflurane for surgery. 2 × 2.5 or 2 × 3 mm craniotomies were performed above the olfactory bulbs and custom cut double windows were implanted. A customized stainless steel head plate was glued directly on the skull posterior to the window, and two stainless steel screws (Neuroscience *Invivo* Research Components) were placed posterior to the head plate. Metabond was then added to cover all exposed skull and a thin layer built to cover the screws and the central surface of the headplate. The position of the craniotomy was biased toward either the left or right bulb.

### 2.3. *In vivo* Imaging

Widefield fluorescent microscopy was used for awake, head-fixed imaging in Thy1-GCaMP6f-GP 5.11 (IMSR Cat# JAX:024339, RRID:IMSR_JAX:024339) mice to view neural activity in the dorsal OB. Mice were between 11 weeks and 13 months old when imaged. 488 nm LED stimulation was used for the duration of the trial (~30 s), but was absent during intertrial intervals (~5 s) to avoid excessive bleaching. All mice were imaged at 30 Hz with 4 × 0.13 NA objective (Nikon). Neural activity was recorded using a Teledyne Photometrics Prime 95B sCMOS camera. For each session, mice were head-fixed above a freely rotating, circular track, allowing mice to run at will during imaging sessions.

### 2.4. *In vitro* OB Slice Imaging

**Figure 2:**
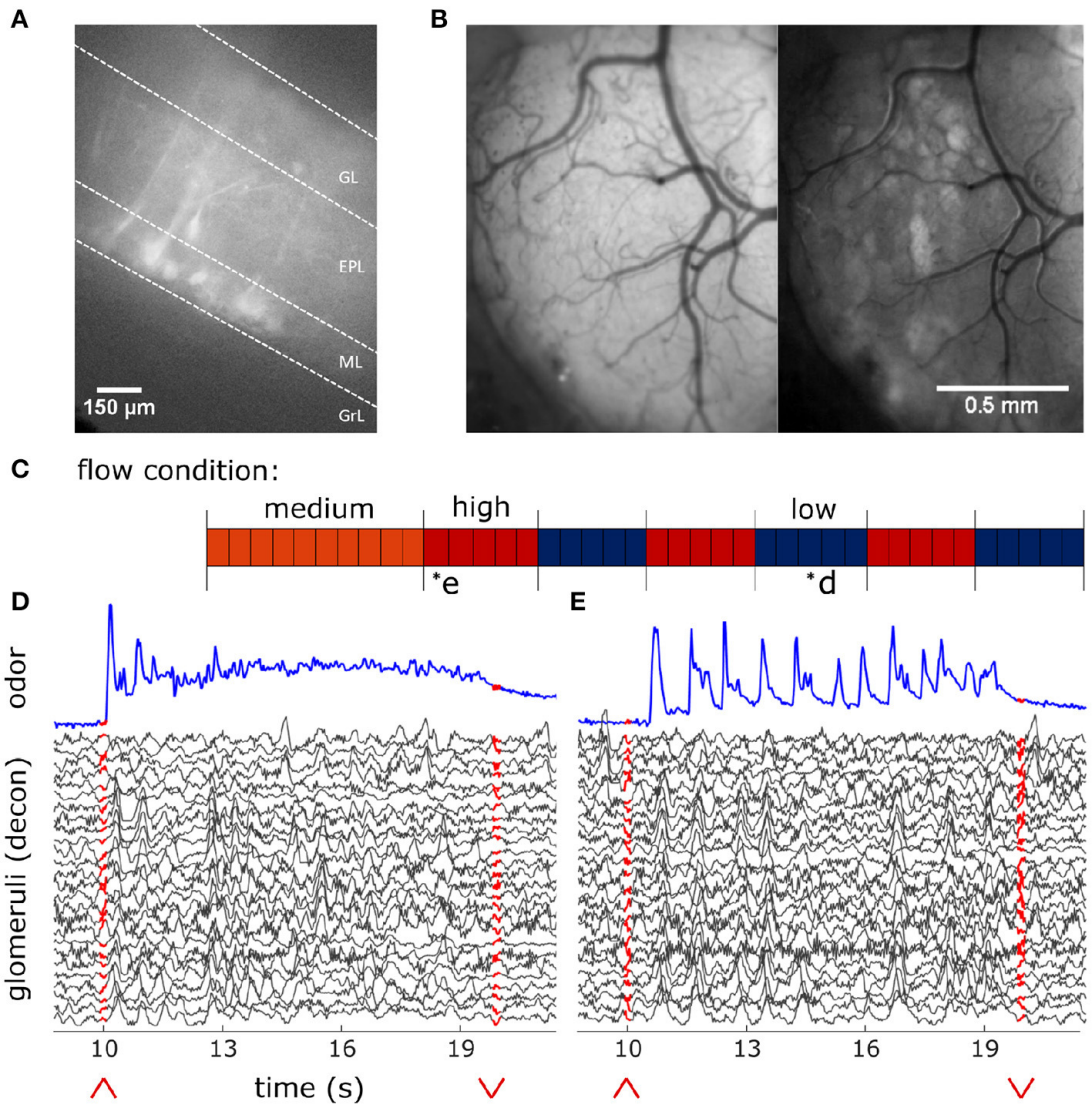
*In-vivo* recording of glomerular population response. **(A)** Change in fluorescence of MT cells in an acute *in vitro* OB slice preparation averaged over 3 s following a puff of high K+ solution. **(B)** *In vivo* view of the dorsal olfactory bulb through an implanted cranial window. (Left) Window activity averaged across a single trial. (Right) Projected standard deviation for the same trial shows MT activity in the dorsal OB responsive to the odor presentation. **(C)** Diagram depicting flow conditions (high, medium, or low) of the 40 trials within a single session. **(D)** The deconvolved ethanol trace (blue) compared to the deconvolved response of each glomeruli (black) within the recorded FOV during a single low flow trial depicted by asterisk in **(C)**. Red arrows indicate onset and offset of plume presentation. **(E)** Same as **(D)** but for a single high flow trial from the same session also depicted by asterisk in **(C)**.

To establish patterns of expression and signals obtained from the OB of Thy1- GCaMP6f- GP 5.11 animals, imaging experiments were conducted in OB slices. These animals show GCaMP6f expression in the main olfactory bulb as well as other areas of olfactory cortex, including but not limited to piriform and anterior olfactory cortex (Dana et al., 2014). Slice work verified strong expression in MT somas and dendrites (Figure 2A). Although we believe MT cells to drive the signal imaged in the OB, it is possible centrifugal feedback could contribute to some of the observed responses.

Horizontal OB slices (300–400 μm) were made following isoflurane anesthesia and decapitation. Olfactory bulbs were rapidly removed and placed in oxygenated (95% O_2_, 5% CO_2_) ice-cold solution containing the following (in mM): 83 NaCl, 2.5 KCl, 3.3 MgSO_4_, 1 NaH_2_PO_4_, 26.2 NaHCO_3_, 22 glucose, 72 sucrose, and 0.5 CaCl_2_. Olfactory bulbs were separated into hemispheres with a razor blade and attached to a stage using adhesive glue applied to the ventral surface of the tissue. Slices were cut using a vibrating microslicer (Leica VT1000S) and were incubated in a holding chamber for 30 min at 32°C. Subsequently, the slices were stored at room temperature.

Slices were placed on a Scientifica SliceScope Pro 6000, using near infrared imaging for slice placement and 488 nm LED illumination for imaging activity and a QI825 Scientific CCD Camera (Q Imaging) for image acquisition. Imaging was performed at 32–35°C. The base extracellular solution contained the following: 125 mM NaCl, 25 mM NaHCO_3_, 1.25 mM NaHPO_4_, 25 mM glucose, 3 mM KCl, 1 mM MgCl_2_, and 2 mM CaCl_2_ (pH 7.3 and adjusted to 295 mOsm), and was oxygenated (95% O_2_, 5% CO_2_). An elevated KCl solution (equimolar replacement of 50 mM NaCl with KCl in the extracellular solution) locally applied through a borosilicate pipette using a picospritzer 2 (Parker Instrumentation) was used to stimulate cells.

### 2.5. Data Pre-processing

ImageJ was used to crop fields of view (FOVs) for data analysis. It was also used to extract pixel averaged signal for hand-drawn region of interest (ROI) analysis.

Matlab 2019b was used to analyze data and plot figures. Data was aligned using NoRMCorre software to perform piecewise rigid and non-rigid motion correction (Pnevmatikakis and Giovannucci, 2017). This alignment corrected for both global frame movement due to head jitter (rigid) and localized distortion due to brain movement (non-rigid).

### 2.6. Precise Tracking of Plume Dynamics

In the past it has been difficult to simultaneously record neural responses and plume dynamics without disrupting plume structure. Photoionization detectors (PIDs) are used to detect odorants, but PIDs sample via an active process, redirecting airflow into the sensor to detect odorants. In these experiments we used a miniaturized ethanol sensor modified from a commercially available metal oxide (MOX) sensor (Tariq et al., 2021). The sensor was placed within 4 mm of the lateral edge of the mouse's right nostril to capture the odor concentration signal across plume presentations for each trial. The sensor, a Figaro TGS 2620 Organic Solvent Vapor Sensor, was adapted similar to described in Tariq et al. (2021) by removing most of the metal head cap, including both a mesh covering and a solid metal covering. The resulting sensor was open to make direct contact with the airflow. In this adapted design, the sensor is mounted only to a circular base plate with a shortened metal cylindrical wall surrounding it. A single odor, a benzaldehyde-ethanol mixture, was used for each trial in the flow experiments. Odors released together travel together within plumes at sufficiently small scales because dispersion dominates over diffusion (Yeung and Pope, 1993; Celani et al., 2014). In this way, the ethanol sensor measured the odor concentration of the benzaldehyde-ethanol mixture. The plume for each trial was released by an automated odor port at the upwind end of the wind tunnel, and the ethanol sensor measured the odor concentration of the ethanol across each trial. During plume presentations, the odor released from the odor port traveled in stochastic plumes through the wind tunnel. Therefore, plume onset time varied on each trial dependent on when the first filament made contact with the plume sensor. Reynold's numbers calculated within each flow condition (see Olfactory Stimuli above) show that plume dynamics are highly unsteady and dynamic, and fully turbulent at both medium and high flow. The odor was released 10 s after the trial began for a duration of 10 s. Each indicated flow condition was maintained throughout the entire trial. Flow condition was set for each trial block (Figure 1C) by adjusting the strength of a vacuum exhaust at the downwind side of the wind tunnel to one of three levels, low, medium, and high. Plumes were presented at medium level flow for the first 10 trials, after which flow alternated between high and low flow in blocks of five trials. Some sessions transitioned from medium to low flow initially and others transitioned from medium to high flow.

### 2.7. Ethanol Deconvolution

Recent characterization of MOX sensors comparing their deconvolved signals to simultaneously recorded PID (200B: mini photo-ionization dectector) signals have validated the use of MOX sensors in capturing turbulent plume dynamics despite their slower recording dynamics (Martinez et al., 2019; Tariq et al., 2021). Tariq et al. show resolution of frequencies up to 15 Hz and high correlations between deconvolved MOX sensor and PID recordings at distances extending to >1 m in a turbulent airflow setting. For our experiments, ethanol concentration throughout each trial was measured by a modified, commercially available ethanol sensor placed within 3.5–4 mm from the mouse's right nostril (Figure 1B). A single session consisted of 40 trials of odor presentation (plumes).

Sensor signal was acquired at 100 Hz and then low pass filtered at 30 Hz using a Kaiser window. The signal, *e*, was then normalized within each trial using the mean and standard deviation of the signal during the plume presentation. The signal was deconvolved by adapting the deconvolution specified in Tariq et al. (2021). The kernel was defined in the same manner, but instead of normalizing the range of the kernel, the integral of the kernel is normalized. Thus, the kernel, *k*, is calculated as follows:

(1) $$\begin{array}{l} k_{0}\left(t\right)=e^{-t/\tau_{decay}}-e^{-t/\tau_{rise}} \end{array}$$

(2) $$\begin{array}{l} k\left(t\right)=k_{0}\left(t\right)/\int_{0}^{T}k_{0}\left(s\right)ds \end{array}$$

where *t* is an array with evenly spaced timestamps at the proper sampling rate for the length of a single trial (T), τ_*decay*_ = 0.4629, and τ_*rise*_ = 0.0001. Both signals, *e* and *k*, are then transformed into Fourier space using the Matlab Fourier transform function, and the ethanol signal is deconvolved in Fourier space by dividing $\hat{e}$ by $\hat{k}$. The inverse Fourier transform of the resulting deconvolution is taken to obtain *d* such that *d* = *F*^−1^($\hat{e}$/$\hat{k}$). The deconvolved signal, *d*, is then normalized within each trial using the mean and standard deviation during the plume presentation, *d* = (*d*− < *d* >)/*std*(*d*). The deconvolution optimizes the preservation of odor concentration dynamics across trials, but does not preserve the absolute value of odor concentration.

To optimize the parameters for the deconvolution, a complete session of 40 plume presentations was recorded with the usual ordering of flow condition blocks (Figure 1C). No mice were recorded during this session. Instead, a PID was placed 4 mm from the ethanol sensor in the same position where the animal is usually head-fixed.

To optimize parameters, the PID signal, *p*, was first downsampled to 100 Hz to match the sensor sampling rate. Next, the signal was normalized within each trial using the mean and standard deviation of the signal during plume presentation, *p* = (*p*− < *p* >)/*std*(*p*). This normalized signal was then Fourier transformed, convolved with the kernel and back-transformed to obtain the convolved signal $c=F^{-1}\left(\hat{p}\cdot \hat{k}\right)$. It was then normalized and compared to the raw ethanol across a range of τ_*decay*_ and τ_*rise*_ parameter values. The Kernel parameters were chosen by minimizing mean squared error between the *e* and normalized *c* signals averaged across all trials within the paired recording session: $\underset{\tau_{rise},\tau_{decay}}{\min}<\|e-\left(c-<c>\right)/std\left(c\right){\|}^{2}>$. This optimized kernel is used to deconvolve the raw ethanol signal in the recording sessions. The deconvolved ethanol sensor signal allows for the recovery of plume dynamics unique to each trial (Supplementary Figure 2). It is significantly correlated with the PID signal as measured during plume presentations (*r* = 0.61, *p* < 0.001, both sampled at 100 Hz), which is a 0.22 improvement from the correlation between the raw ethanol sensor and PID signal (*r* = 0.39, *p* < 0.001, both sampled at 100 Hz).

Finally, with the exception of Supplementary Figure 1, the deconvolved trace was downsampled for figures and analyses to match the calcium trace (30 Hz) by averaging all samples taken across each camera frame. Deconvolution of the sensor signal during paired recordings is plotted and compared to both the raw sensor signal and to the PID reading from the paired recordings (Supplementary Figure 1). An initial inflection of signal at plume onset can be observed in the deconvolved ethanol signal for some trials (Figure 1D, Supplementary Figure 1B). This peak at plume onset is not reported by the raw sensor signal (Supplementary Figure 1A) or by the PID signal (Supplementary Figure 1C) and is likely an artifact of the deconvolution. Since these experiments focus on how well MT activity follows odor concentration dynamics during plume encounters, the first and last seconds of the 10 s plume are omitted when analyzing neural responses to plume dynamics. The only exception is for the analysis of responsivity, which is based on the percentage of timepoints for which a significant response is observed and so this thresholded measure does not directly consider signal magnitude. Therefore, any artifact of plume onset dynamics in the sensor signal due to the deconvolution of its slower dynamics do not affect correlations reported between stimulus and response.

### 2.8. Defining Dynamic Flow

Dynamic flow was calculated within each session. In the experimental setup, flow condition is defined by wind speed. Intermittency was measured within a trial during the middle 8 s of the 10 s plume presentation by calculating asymmetry or by using the 3rd moment of the sampled distribution (the distribution odor concentration magnitude as measured for each time point across the window) (Figure 1E). Low and high flow trials were separable using either of these measures. As determined by these measures, intermittency increased with airflow moving from low to high flow conditions. The PID signal from the paired odor recording session shows this difference in dynamics as the concentration plotted across high flow trials exhibits increased intermittency as compared to that for low flow trials (Supplementary Figure 1C).

To see if there was a main effect of flow condition on these stimulus properties, skewness and asymmetry, a one-way analysis of variance (ANOVA) test was conducted for each parameter. For each ANOVA, multiple comparisons using a Tukey's test were performed to look for significant differences of the parameter between flow where comparisons were considered significant for *p* < 0.01. These tests indicated that both skewness and asymmetry vary significantly across flow condition [*F*_(2, 155)_ = 52.7, *p* < 0.001 and *F*_(2, 155)_ = 54.9, *p* < 0.001, respectively]. Multiple comparison tests examining the differential effect of flow on these parameters showed that, for both, there was a significant difference between low and medium flow and a significant difference between low and high flow. No significant difference was found between medium and high flow. Therefore, only low and high flow conditions were selected when examining the effect of air flow on neural parameters.

### 2.9. Measuring Glomerular Responses to Plume Dynamics

Widefield imaging of the dorsal surface of the OB in Thy1-GCaMP6f-GP5.11 mice was used to capture MT cell activity at the glomerular level (Figure 2B). Thy1 mice exhibit fast kinetics and strong expression in MT cells within the OB (Dana et al., 2014). Global MT activity is clustered into dendritic complexes known as glomeruli. Widespread activity of secondary dendrites in the external plexiform layer (EPL) of the dorsal OB causes diffuse fluorescence across the imaging field. Therefore, CaImAn, a constrained non-negative matrix factorization (CNMF) algorithm, was used on each FOV to find regions of interest (ROIs) (FOV 1, FOV 2, and FOV 3 from mouse 1, *n* = 27, 17, and 6 glomeruli, respectively; FOV 4 from mouse 2, *n* = 35; FOV 5 from mouse 3, *n* = 26) and their activity traces (Pnevmatikakis et al., 2016). Thus, the spatial decomposition of CNMF provided the ROI for each glomerulus, and the temporal decomposition provided its corresponding denoised activity trace. The denoising of the CNMF temporal decomposition helps to remove correlated signals between neighboring glomeruli, accounts for calcium drift within recording sessions, and separates glomeruli overlapping in the dorsal-ventral dimension. The change in the distribution of correlation coefficients between ROIs before and after denoising (Supplementary Figure 3) shows a decorrelation of glomerular signals. A two-sample Kolmogorov-Smirnoff test shows the distributions of correlation coefficients between the pixel averaged ROI traces, 0.87 ± 0.07 (mean ± st. dev), and the CNMF ROI traces, 0.36 ± 0.28 (mean ± st. dev), are significantly different (*D* = 0.86, *p* < 0.001).

To protect against over-segmenting a single glomerulus into multiple ROIs, neighboring ROIs whose baseline CNMF activity was correlated above 0.75 were selected as candidates for ROI merging (Supplementary Figure 4). The baseline period was examined as criteria for possible merging since glomeruli might have similar response profiles to the stimulus dynamics during plume presentations. The baseline activity of the neighboring ROIs was then binarized using a threshold of ± 1 st. dev. If the correlation of the binarized activity between neighboring ROIs exceeded 0.75, the ROI with the lower mean activity (presumably encompassing less of the glomerulus) was dropped from the analysis.

To validate the use of CNMF for ROI selection, results were compared to a hand-drawn ROI analysis conducted on one of the fields of view (FOV 5) in ImageJ (Supplementary Figure 5). Using this standard, manual ROI selection, we show CNMF decomposition and denoising does not qualitatively change the interpretation of the data. Rather, CNMF recovers spatial and temporal resolution of MT activity by increasing the number of ROIs detected per field and decreasing pairwise correlation between ROIs. This decrease in pairwise correlation between ROIs suggests CNMF reduces common global signal, such as that from neuropil activity where secondary dendrites and global centrifugal feedback in the OB produce diffuse excitation across the dorsal surface of the OB. Hand-drawn ROIs were selected after viewing footage and reviewing standard deviation and maximum value projections of activity from the FOV within each trial. The activity averaged from within hand-drawn ROIs has higher pairwise correlations than denoised CNMF activity traces. This mirrors what is seen when pixel-averaged activity from within CNMF ROIs (without denoising) is compared to the denoised traces. Thus, the denoising of CNMF recovers the spatiotemporal resolution of glomerular activity observed in the dorsal OB recordings in both types of analyses. A two-sample Kolmogorov-Smirnoff test shows the distributions of correlation coefficients between the deconvoled hand-drawn ROIs, 0.81 ± 0.08 (mean ± st. dev), and the deconvolved CNMF traces, 0.1935 ± 0.3471 (mean ± st. dev), are significantly different (*D* = 0.86, *p* < 0.001). Results of the hand-drawn ROI analysis (power and correlation analyses, Supplementary Figures 5D,E, respectively) were qualitatively similar to those found using CNMF corroborating the ability of glomerular networks to resolve odor concentration dynamics. Cross-correlations show a relation between glomerular and ethanol signals during odor presentation with all glomeruli having significant correlation with the plume during odor presentation as compared to their respective null distributions from trial shuffled correlation analyses. In addition, a strong correlation between glomerular response power (0–5 Hz) and ability to track odor concentration dynamics is also present in the hand-drawn ROIs (*r* = 0.80, *p* = < 0.001). Thus, we find that CNMF captures the relationship between glomeruli and plume dynamics while improving the resolution of glomerular network activity and inter-glomerular temporal dynamics.

The CNMF activity traces from the identified glomeruli were baseline normalized using the mean and standard deviation of a 5 s baseline activity period prior to stimulus onset. Traces for each glomerulus were then deconvolved in the style of Stern et al. (2020) to recover the average activity rate of each glomerulus. To find the optimal penalty parameter, λ, for deconvolution, λ was optimized within each glomerulus. Then the median of this optimized distribution was used as the λ in the deconvolution for all glomeruli. After deconvolution, traces were standardized using the standard deviation of the glomerulus's entire trace. Deconvolved signals were standardized in this way since the florescence range of a glomerulus's response depends on the number of expressing MT cells, the depth of the glomerulus from the dorsal surface, and other methodological factors unrelated to the magnitude of the response.

### 2.10. Testing for Responsive Glomeruli

A glomerulus was considered to be responsive to odor if its deconvolved trace exceeded threshold more time points than expected by chance during plume presentations as compared to its activity level during odorless baseline periods (**Figure 6**). Since this preliminary measure does not rely on stimulus dynamics, it captures glomeruli that respond to the plume even if their response is unrelated to odor concentration dynamics or only present for part of the plume.

First, the deconvolved trace of a single glomerulus was split into two periods: baseline activity and odor response. The baseline period is a 5 s period at the beginning of each trial prior to plume onset. The odor response period is the time during which the plume is present as well as 1 s immediately following the plume since inhibition has been shown to induce excitatory rebound responses in tufted cells (Cavarretta et al., 2018). The signal is first baseline normalized by subtracting the mean and dividing by the st. dev of the baseline activity within each trial. Next, it is binarized, thresholding for time points where activity exceeded the 95% confidence interval of the glomerulus's original baseline activity (thresholded at ± 1.96 baseline mean). In this way, each time point that crossed the threshold was considered an event. Within each trial's plume presentation, if the number of events exceeded the null expectation (5% of the total number of time points during plume presentation rounded up to the nearest integer), the glomerulus was considered to be responsive to the plume during that trial. The proportion of trials to which the glomerulus responded was calculated within three sets of flow conditions (all flows, low flow, and high flow). To illustrate responsivity scores within and between glomeruli simultaneously, scores are plotted as a stacked bar graph (**Figure 6A**, cumulative scores are not used for analytic purposes).

### 2.11. Cross-Correlation Between Plume Dynamics and Corresponding Neural Responses

To understand the relation between stimulus and response time series, a preliminary analysis was conducted by calculating the correlation coefficients between the two signals for each glomerulus. In the future, more sophisticated techniques will be used to establish how much of the neural representations can be explained by high-fidelity odor concentration encoding.

Due to the stochastic nature of plume onset and offset times, the correlation is only calculated for the middle 8 s of the 10 s plume so that onset and offset dynamics are not included and the correlation measure represents the magnitude of plume tracking during plume encounters. The cross-correlation coefficient of a glomerulus *r*_*g*_ is calculated between the ethanol *e* and calcium *c* deconvolutions during plume presentations. For a single glomerulus, the correlation coefficient between the two deconvolutions within a single trial *n* is calculated at all possible lags *l*. Using the xcorr() function in Matlab to compute the coefficients, both signals are mean subtracted prior to calculating the cross-correlations such that the correlation coefficients are synonymous with calculating the Pearson correlation coefficient between the two signals at each respective lag value.

(3) $$\begin{array}{l} r_{g,n,l}=corr\left(e_{n,l},c_{n,l}\right) \end{array}$$

The mean coefficient for each glomerulus, ${\bar{r}}_{g,l}$, is calculated by averaging across all trials within the session (*n* = 40 for 3 FOVs, and *n* = 39 for 2 FOVs) at each possible lag. The maximum coefficient mean is selected from all lags within a 500 ms window *w* of the neural activity following the ethanol signal.

(4) $$\begin{array}{l} r_{g}=\underset{0<l\leq w}{\max}{\bar{r}}_{g,l} \end{array}$$

This is considered to be a window of sufficient size to account for variable delays in glomerular processing. The average time lag of *r*_*g*_ was 130 ms ± 100 (mean ± st. dev).

Within flow cross-correlations are calculated in the same manner but averaged only across trials within the specified flow condition.

For plotting of tracking ability (**Figures 5A,B**), *r*_*g*_ is compared to a single trial shuffled analysis using the same method as detailed above. The difference between the matched and shuffled coefficients suggest correlations are not solely a result of plume structure, but are driven by the temporal dynamics unique to each trial. Trials were shuffled within each glomerulus by calculating the correlations between *e*_*n*_ and *c*_≠*n*_. In this way, any relation dependent on the dynamics of the stochastic fluctuations within each plume presentation is lost, but other statistical features of the plume presentation are preserved, yielding a baseline value for the cross-correlation. Glomeruli are plotted in **Figures 5A,B** if their correlation coefficient from the matched analysis exceeds ± 2 standard deviations (st. dev of the coefficient distribution from the shuffled analysis) of the shuffled mean coefficient. Since correlation varies significantly within a glomerulus across flow conditions, a glomerulus is considered to exceed the shuffled mean if it does so in at least one of the three defined conditions, all flows, low flow, or high flow.

Using the same shuffled correlation, a bootstrap analysis was conducted (10,000 iterations) creating a null distribution of the shuffled mean correlation coefficients to test for significance (Supplementary Figure 7C). The mean correlations are compared to their respective 95% confidence interval for the null distribution. Glomeruli are considered to respond significantly to plume dynamics when their mean coefficients exceed their null expectation. Non-significantly responding glomeruli are depicted in stacked bar graphs using gray hues (**Figures 5C**, **6A**).

Comparison of correlation coefficients in the matched vs. shuffled cross-correlations does not naturally divide the glomeruli into two subpopulations, but rather the strength of this relationship varies continuously across glomeruli. Therefore, instead of dividing glomeruli into subpopulations of tracking vs. non-tracking, our analyses consider how the strength of odor concentration tracking compares to other properties of the glomerulus and its response.

## 3. Results

### 3.1. Measuring Glomerular Responses to Plume Dynamics

Using a modified, commercially available odor sensor combined with widefield calcium imaging techniques in head-fixed mice, we reliably tracked plume dynamics and investigated glomerular responses to this fluctuating input. Imaging was conducted in Thy1-GCaMP6f-GP5.11 mice which have fast kinetics and expression in mitral and tufted (MT) cells within the olfactory bulb (OB) (Dana et al., 2014) (Figure 2A). Widefield imaging of the dorsal surface of the OB allows for glomerular level resolution of the neural response (Figure 2B) (Fletcher et al., 2009). To explore a range of plume dynamics an animal may encounter in its natural environment, we changed the airspeed in the wind tunnel to create stochastic plumes with different odor concentration dynamics (Figures 1D–H). Odor identity, concentration and volume released from the odor port remained constant across all flow conditions, making the plume dynamics the only source of variation (Figures 2C–E).

### 3.2. Mitral and Tufted Population Activity Correlates With Plume Dynamics

**Figure 3:**
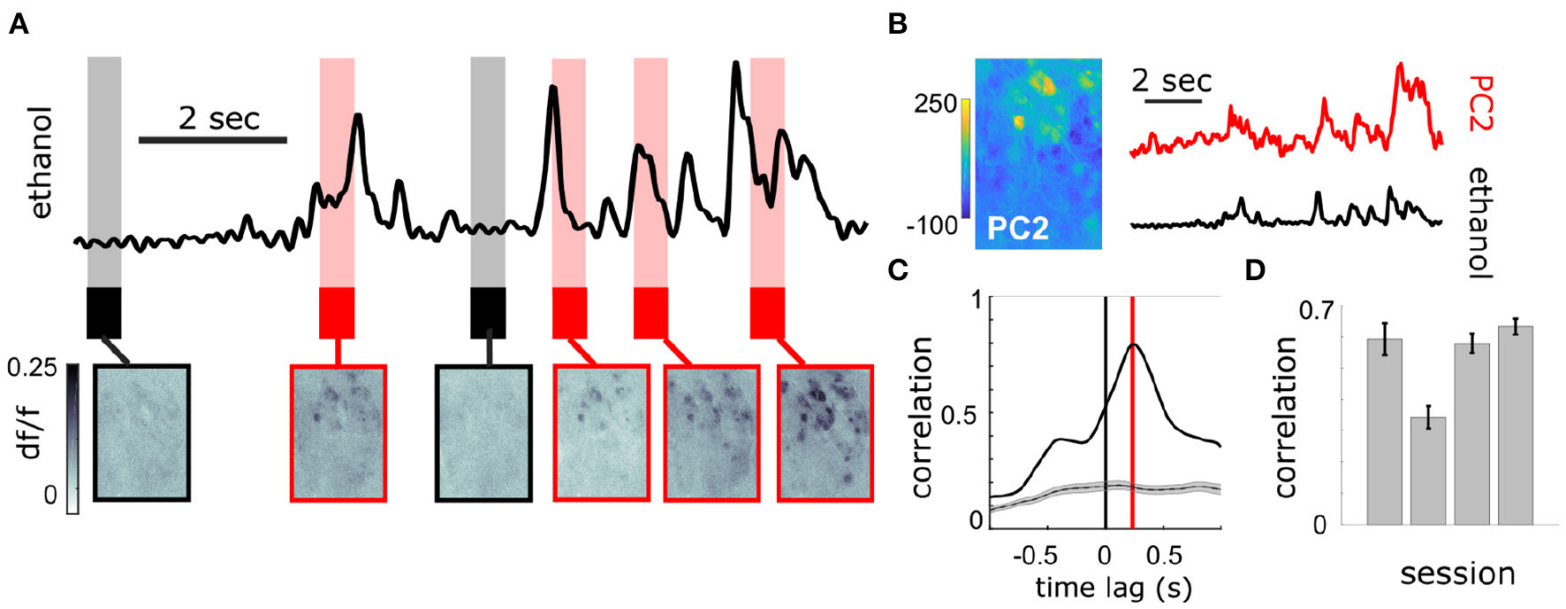
Population response of MT cells in dorsal OB respond to changes in odor concentration during plume presentations. **(A)** Simultaneously recorded deconvolved ethanol plume (top) and imaging of calcium signals from MT cell activity in an example FOV of a Thy1-GCaMP6f (GP5.11) mouse (bottom). Baseline and odorless periods (black) and odor plume input (red) are shown from the indicated time points. Fluctuations in the odor plume elicit repeatable activation of specific glomerular networks in response to whiffs of odor during plume presentations. **(B)** (Left) An image of the principal component loadings corresponding to the odor-evoked activity [principal component 2 (PC2)]. (Right) Time series of PC2 (top, red) aligned to the simultaneous ethanol signal (bottom, black). Scale bar indicates 2 s. **(C)** Cross-correlogram between the two signals in **(B)**. Red line indicates a slight offset from 0 for the peak correlation (~250 ms mean lag across FOVs from sensor to OB response). Gray plots average the null correlation ± SEM (correlation of neural activity from the example trial with odor signal from all non-matched trials in session). **(D)** Cross-correlations (mean ± SEM) between odor evoked population activity (principal component) and ethanol sensor signal are strong across 3 Thy1-GCaMP6f (GP5.11) mice (*r* = 0.54 ± 0.07).

At the bulbar level, imaging of MT cell activity shows activation of glomerular networks during odor exposure (Figure 3A). The global MT activity for a given field of view (FOV) was subjected to principal component analysis (PCA) and compared to the simultaneously recorded plume dynamics (Figure 3B). The FOV's were aligned prior to PCA, but no segmentation or denoising was performed. To search for component activity responsive to plume dynamics, the correlation between each principal component and the odor concentration dynamics was calculated. There exists a high ranking component for each mouse that correlates strongly with plume dynamics (Figures 3C,D). Plotting the loading weights of the maximally correlated component shows dense clusters of high variance resembling partial spatial maps of glomerular activity. These findings demonstrate that MT population activity recorded in the first relay of olfactory processing is correlated to odor concentration dynamics during plume presentations. In order to establish whether individual glomeruli are correlated to odor cues, we sought to segment the MT activity into glomerular units to determine their respective contributions to the observed tracking of plume fluctuations by population activity.

### 3.3. Neural Activity of Glomeruli

**Figure 4:**
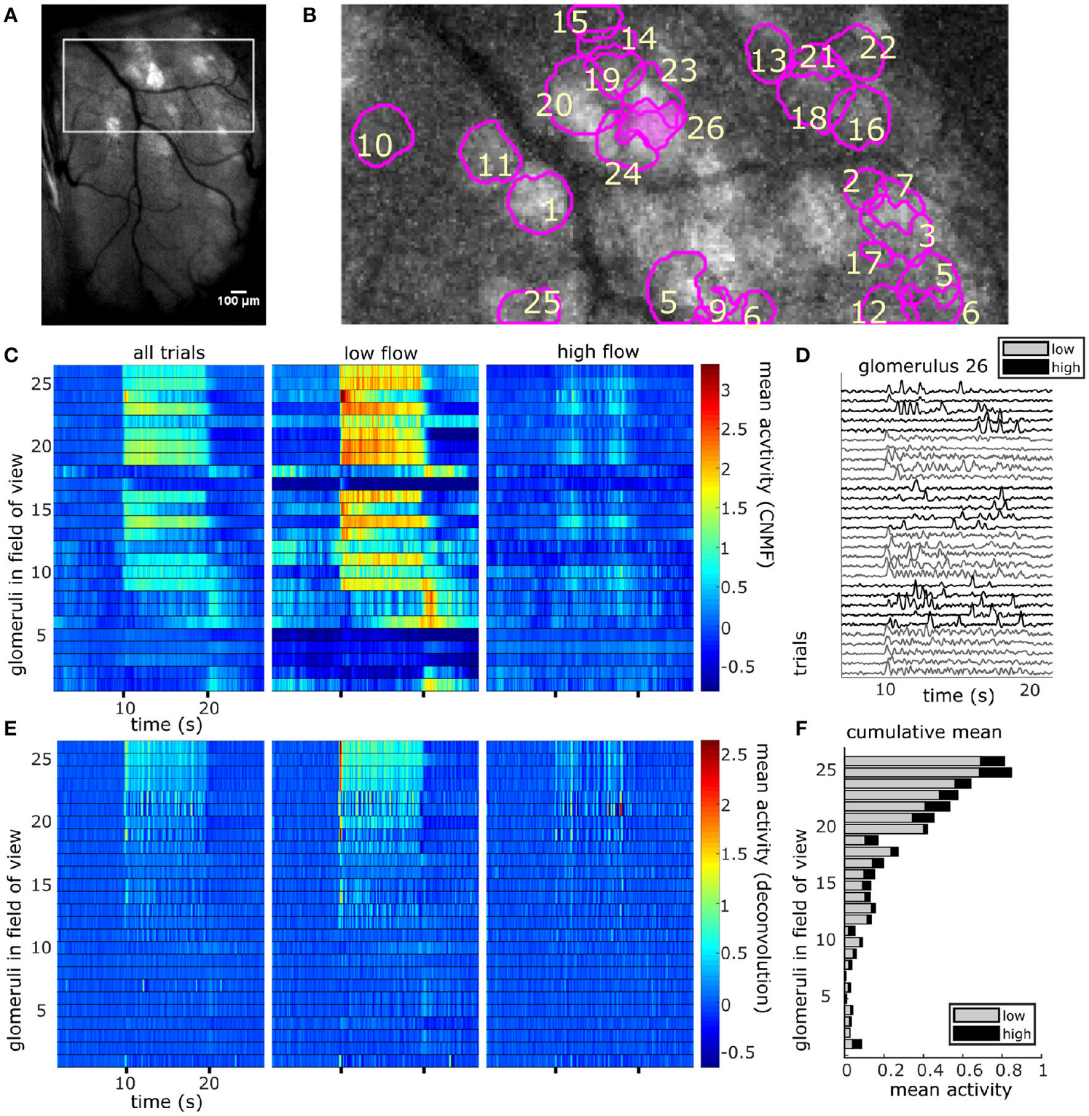
The spatial and temporal decomposition of CNMF identifies glomeruli and denoises their traces. **(A)** The white box outlines the FOV used for analysis as it relates to the larger recording window. The image shows the standard deviation projection of the aligned recording during a single odor presentation. **(B)** Mean subtracted maximum projection of the same trial overlaid with ROIs from CNMF spatial decomposition shows segmentation of glomeruli for a single FOV using CNMF spatial decomposition. The spatial decomposition of the FOV results in 26 glomeruli (four dropped units after merge analysis not pictured) as outlined and numbered. **(C)** Shows the mean traces of each glomerulus's CNMF temporal decomposition within each flow condition (left to right : all trials, low flow, high flow). Trials sorted by magnitude of normalized mean deconvolved response **(E)** during odor exposure. **(D)** The deconvolved CNMF response of a single glomerulus [pink fill **(B)**] to all low (gray) and high (black) flow trials across the recording session shows glomerular responses vary due to the unique odor concentration dynamics of each plume. **(E)** Deconvolution accelerates dynamics of glomerular responses as shown by the mean deconvolved traces of the corresponding glomeruli depicted in **(C)**. **(F)** The cumulative mean, a sum of the mean responses for each glomerulus in low and high flow, are plotted as a stacked bar graph so that comparisons between mean responses can be made within and across glomeruli simultaneously. Mean responses are calculated for the deconvolution **(E)** within each flow condition during the plume release and vary significantly between conditions [*t*_(110)_ = 11.43, *p* < 0.001] with higher average responses in low flow.

CNMF decomposition provided locations of glomeruli and their corresponding denoised traces (Figure 4). To recover the average activity of synaptic complexes of MT activity known as glomeruli, CNMF traces were deconvolved in the style of Stern et al. (2020) (see methods). The mean deconvolved trace across trials was calculated for each glomerulus during plume presentations (Figure 4E). The mean response of a deconvolved trace (Figure 4F) was only considered during the middle 8 s of the 10 s odor plume to concentrate on glomerular responses to odor concentration dynamics during plume presentations and avoid responses to onset or offset plume dynamics.

For each glomerulus, the mean response across plumes was also calculated within low and high flow conditions. A paired samples *t*-test found that glomerular response means varied significantly across low and high flow conditions [*t*_(110)_ = 9.71, *p* < 0.001]. Mean responses were higher in low flow conditions, during which lower airspeed resulted in plume dynamics that were less intermittent, as shown by lower skewness and asymmetry in the deconvolved odor signals of low flow trials as compared to high flow trials [*F*_(2, 155)_ = 52.7, *p* < 0.001 and *F*_(2, 155)_ = 54.9, *p* < 0.001, respectively] (Figures 1E–H). In high flow conditions, increased intermittency produced more brief, high concentration fluctuations followed by blanks, or periods without odor signal. This decreased response observed across high flow conditions could be due to a decrease in odor concentration means as plumes had lower concentration means in high flow. PID recordings from the paired recording experiment show a significant 50% decrease of the mean concentration in high flow as compared to low flow (*z* = 6.65, *p* < 0.001). Thus, the mean MT activity increased in low flow trials following the increase in stimulus mean, but activity became less correlated with plume dynamics, suggesting the response of glomerular populations are moderated by plume dynamics.

### 3.4. Correlation Between Stimulus and Glomerular Activity

**Figure 5:**
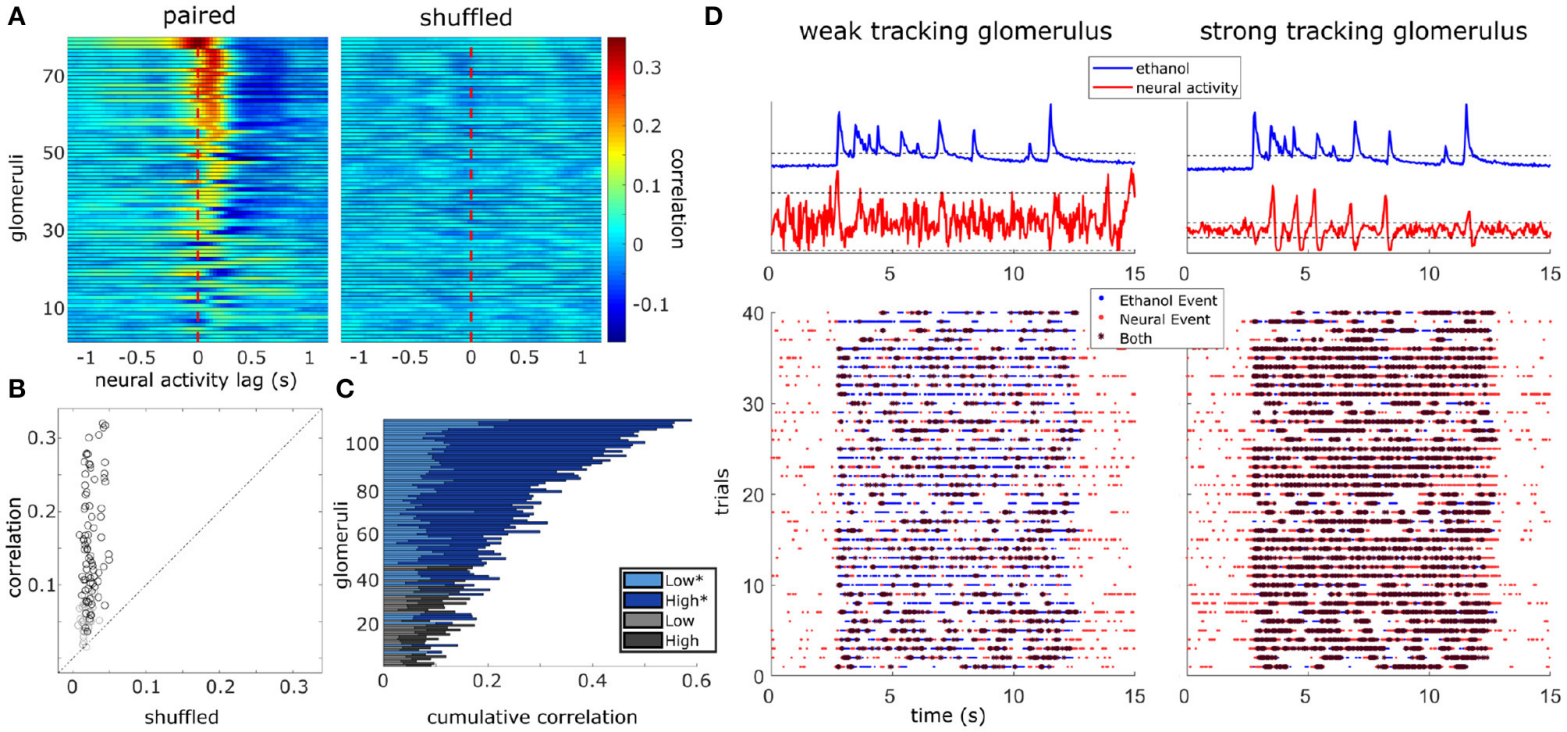
Glomerular population activity follows odor concentration dynamics across plume encounters. (Left) The cross-correlation between the deconvolved ethanol trace and each glomerulus's deconvolved activity trace is calculated within each trial and then averaged across trials. Each row is a glomeruli and each time point represents the cross-correlation at the indicated lag. Glomeruli are sorted in order of decreasing magnitude of correlation coefficient (see methods). (Right) Same as left but glomerular responses are trial shuffled so that the signals compared are not from the same trial. Glomeruli are sorted to match their corresponding unshuffled cross-correlation in the right panel. **(B)** Scatterplot of the correlation coefficient of all glomeruli if compared to their respective shuffled coefficient. Glomeruli plotted in **(A)** are marked in black if their coefficient exceeds their shuffled coefficient from a single trial shuffled comparison by 2 standard deviations. **(C)** The cumulative correlation, a sum of correlation coefficients for each glomerulus in low and high flow, are plotted as a stacked bar graph so that comparisons between mean responses can be made within and across glomeruli simultaneously. The cumulative plotting shows variation in ability to detect changes in odor concentration dynamics across glomeruli both within and across flow conditions. On average, a glomerulus's tracking ability varies significantly between conditions [*t*_(110)_ = 12.81, *p* < 0.001], with most glomeruli having stronger correlation coefficients in high flow trials. Glomeruli that significantly correlate with plume dynamics in at least one condition are plotted in blues (*) while those that do not are plotted in grays. **(D)** Binary cross-correlation. Top: Simultaneously recorded signals shown for two example glomeruli responding to the same example trial's odor plume. Odor and glomerular activity traces plotted with their respective thresholds (dotted, odor threshold: mean during plume presentation, neural threshold: ± 2 st. dev of baseline). Bottom: Resulting binarized traces plotted for each trial illustrate the magnitude of concurrent activity as events (stars) between the plume and the response of each glomerulus across the experimental session.

To determine if plume dynamics could be moderating the glomerular population response, cross-correlation was used to quantify the relation between odor concentration dynamics and simultaneously recorded glomerular activity (Figure 5). Most glomeruli significantly followed plume dynamics when correlation between neural activity and odor activity was calculated across all trials (100/111), across low flow (97/111) trials, or across high flow (100/111) trials. Significant tracking of the stochastic changes in odor concentration across plume presentations is determined by comparing mean correlation coefficients to a null distribution created using a trial shuffled bootstrap analysis (see methods) (Supplementary Figure 7C). Within glomeruli that significantly responded to plume dynamics, the degree of tracking (the strength of the correlation between plume dynamics and a glomerulus's response) varied along a continuum such that some glomeruli were more responsive to fluctuations in odor concentration than others (Figures 5C,D). Higher correlation coefficients are not observed when glomerular responses are trial shuffled and ethanol recordings are no longer compared to the glomerular responses they elicited (Figures 5A,B, Supplementary Figure 7C). This shows that it is not the statistics of stimulus presentations that drive this correlation, but rather the plume's temporal dynamics unique to each trial.

Correlation coefficients (tracking) increased from the null expectations by 0.13 ± 0.08 (mean ± st. dev) across all flow conditions, 0.08 ± 0.07 within low flow, and 0.16 ± 0.11 within high flow. Glomeruli were significantly better at tracking plume dynamics in high flow than they were in low [*t*_(110)_ = 12.81, *p* < 0.001] (Figure 5C) with average correlation coefficients increasing by 0.11 ± 0.09 (mean ± st. dev). We wondered if this increase in correlation could result from increased sparsity. Indeed correlation between two signals where the values are constant or zero for most of the time is automatically high, even if the peaks are entirely uncorrelated. If this was the case, the shuffled correlations in high flow should be significantly higher than in low flow, but this is not observed when looking at the confidence intervals for the null correlation coefficients computed within flow conditions (Supplementary Figure 7C). Thus, a large fraction of the glomerular population follows fluctuations during plume encounters, and the degree of dynamic tracking is moderated by plume dynamics, becoming stronger on average during plumes with higher levels of intermittency (as measured by increased skewness in high flow trials).

A second set of experiments, an odor panel, was used to assess whether the tracking behavior seen in the flow experiments is generalizable beyond the benzaldehyde mixture used. We recorded MT activity as it responded to plumes of three different odors within a single session. Plumes consisted of either ethanol (without a mixed odorant), benzaldehyde, or isoamyl acetate. For all trials, flow stayed constant and was set to the high flow condition. Results corroborated the ability of MT activity to respond to odor concentration dynamics and show tracking behavior generalizes beyond the benzaldehyde mixture used in the flow experiments. Glomeruli that significantly responded to odor concentration dynamics were found in the ethanol only condition (41/59 glomeruli), the benzaldehyde/ethanol condition (38/59), and the isoamyl acetate/ethanol condition (41/59) (Supplementary Figure 8A), and the majority of glomeruli reached significance in at least one odor condition (54/59).

### 3.5. Plume Fluctuations Structure Glomerular Network Dynamics

We measured glomerular responsivity and response power to see the effect of flow condition on these measures and whether these measures were related to how well a glomerulus followed plume dynamics.

**Figure 6:**
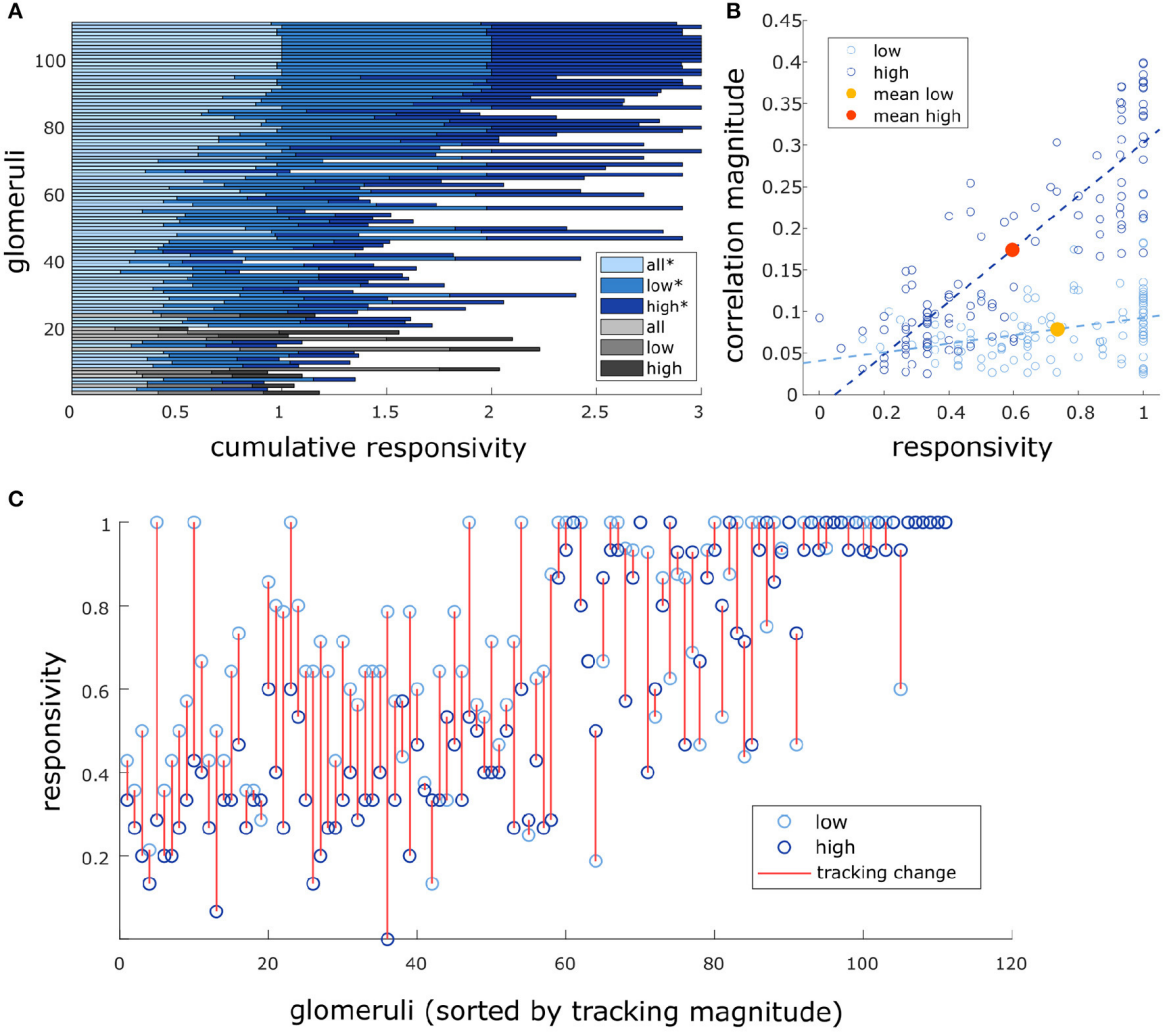
Glomeruli that respond more reliably to plumes are more correlated with their dynamics. **(A)** Responsivity scores plotted as a cumulative bar graph to illustrate differences within and across glomeruli when responsivity is calculated across all conditions (lightest blue) or is calculated exclusively within low flow (medium blue) or high flow (dark blue). Glomeruli are sorted top to bottom by decreasing average tracking ability (correlation magnitude) and glomeruli that significantly track plume dynamics (as defined in methods) are plotted in blue hues(*) while those that do not are plotted in gray hues. The graph shows magnitude of odor concentration tracking is correlated with (*r* = 0.76, *p* < 0.001), but is not strictly defined by response reliability as glomeruli exist that respond strongly to odor presence but not to concentration dynamics. In addition, within flow comparisons show responsivity is significantly higher in low flow than high flow [*t*_(110)_ = 12.1263, *p* < 0.001]. **(B)** Within flow condition, responsivity is plotted against tracking ability (correlation magnitude) for each glomerulus (circle). To represent the population response, the average responsivity across all glomeruli (low average = yellow dot, high average = red dot) is plotted against average correlation with plume dynamics within low (light blue) and high (dark blue) flow conditions illustrating how flow moderates these relationships. Across glomeruli, responsivity is positively correlated with tracking ability as is illustrated by the lines of best fit. On average, higher flow predicts a decrease in average responsivity level but also predicts an increase in tracking ability. **(C)** Average responsivity in low and high flow is plotted for each glomerulus, and the change in mean responsivity between flows shown in **(A)** is explicitly plotted for each glomerulus (red line) as well. Glomeruli are sorted by increasing tracking ability (from left to right) showing glomeruli with higher response reliability are more sensitive to plume dynamics.

We found that there was a significant effect of flow condition on glomerular responsivity. Responsivity is defined as the proportion of trials to which a glomerulus responded to the odor (see methods). Glomeruli had significantly higher responsivity during low flow trials (*t* = 12.1, *p* < 0.001), with responsivity scores increasing by 0.21 ± 0.18 (mean ± st. dev) as compared to high flow (Figure 6). Therefore, glomeruli responded to low flow trials more reliably than they responded to high flow trials. As noted previously, the average correlation between plume dynamics and MT activity increased in high flow conditions, so although glomerular responses became less reliable as airflow increased, they became more correlated with plume dynamics (Figure 6B).

**Figure 7:**
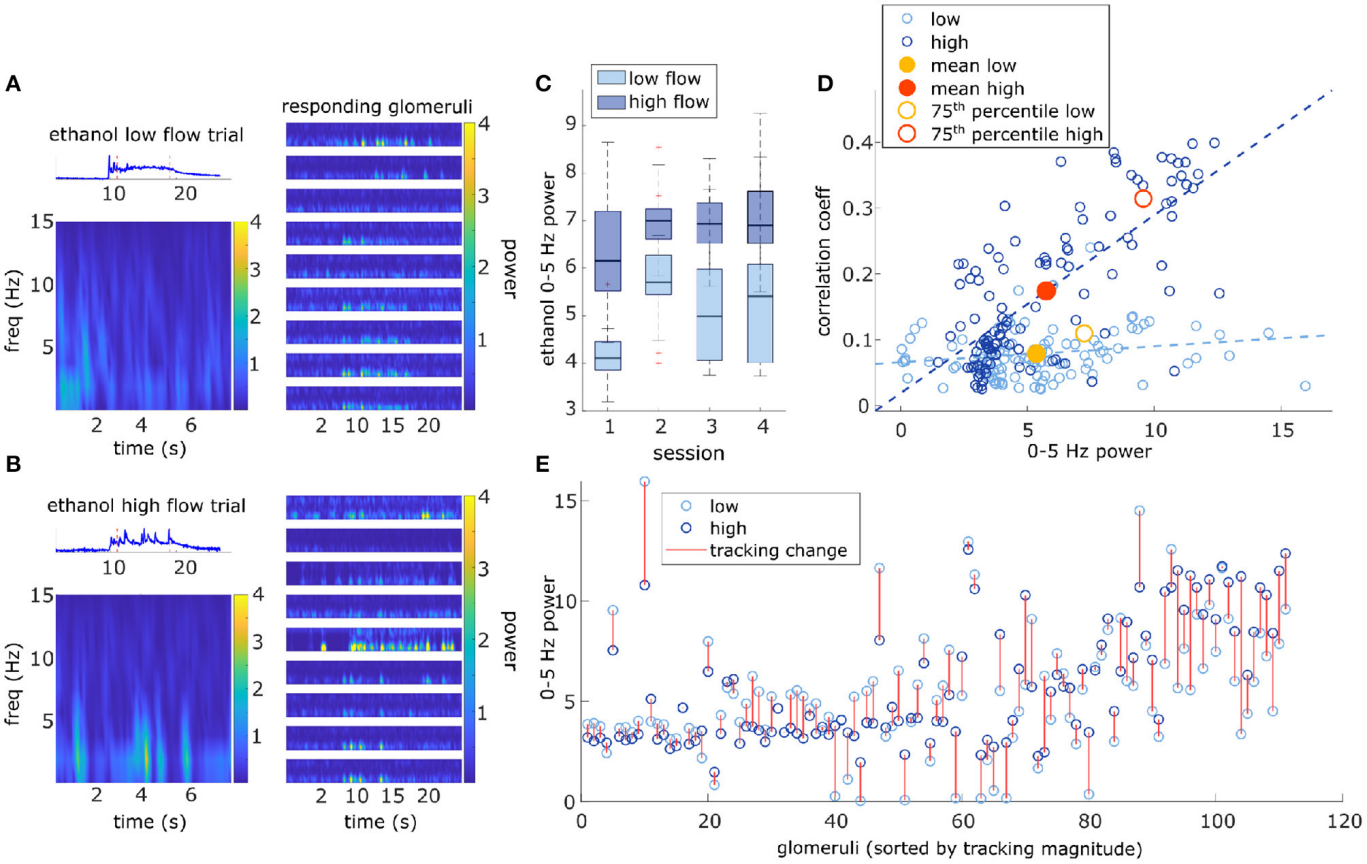
Higher magnitude of glomerular response power (0–5 Hz) is associated with higher correlation with plume dynamics. **(A)** Ethanol signal from a single low flow trial is plotted (top-left) and a corresponding Short-time Fourier transform (STFT) of the plume (time between red dotted lines) is shown below (bottom-left). STFTs are also shown for a sample of glomeruli responding to the plume (right). STFTs show most response power of the glomeruli and odor signal is concentrated between 0 and 5 Hz. Glomeruli STFTs are sorted (top to bottom) by increasing correlation with plume dynamics. **(B)** Same as **(A)** but for a single high flow trial from another example FOV. **(C)** Box plots for the distributions of stimulus power (0–5 Hz) for all trials within the demarcated flow condition are plotted for each session. On average, high flow distribution means (dark blue) significantly exceeded low flow distribution means (light blue) [*t*_(116)_ = 31, *p* < 0.001]. **(D)** Within both high and low flow conditions, tracking (correlation magnitude of glomerular responses with plume dynamics) is plotted against response power (0–5 Hz power spectrum change between “odor off” and “odor on” periods) for each glomerulus (blue hues, circles). Glomeruli with stronger tracking have a greater increase in response power during plume presentations (*r* = 0.74, *p* < 0.001). When calculated within flow, this relationship is significant within high flow (*r* = 0.73, *p* < 0.001), but not within low flow (*r* = 0.19, *p* = 0.05). The average response across all glomeruli is plotted (low average = yellow dot, high average = red dot) to represent the population response. Mean response power of the glomerular population is not significantly different between low and high flow, except for when calculated with glomeruli whose mean activity is in the 75th percentile (low average = yellow circle, high average = red circle). **(E)** Response power of each glomerulus is again plotted, but the change within a glomerulus between low flow (light blue) and high flow (dark blue) is signified by the red line. Glomeruli are plotted sequentially along the x-axis and are sorted left to right by increasing tracking ability. As tracking ability increases, so does the change in response power between flow conditions. This is consistent with the significant change in mean response between flow condition observed in the 75th percentile [plotted as red/yellow circles in **(D)**].

Responsivity is a thresholded measure that determines if a glomerulus is more active than expected by chance during a plume presentation and does not capture the dynamics present in the response. The strength of the dynamic activity of glomeruli was determined by measuring the change in cumulative response power between baseline periods and plume presentations (see methods) (Supplementary Figure 6). Fast Fourier transform was used to measure response power within 0–5 Hz, a frequency range relative to the stimulus dynamics (Figure 7). Response power (0–5 Hz) increased significantly from baseline during plume presentations [*t*_(110)_ = 20, *p* < 0.001] by 5.7 ± 3.0 a.u. (mean ± st. dev), a 448% increase (Figure 7). On average 86.7 ± 4.9% (mean ± st. dev) of cumulative stimulus power for each session was within 0–5 Hz. Stimulus power was measured using the deconvolved odor signal of the plumes to which MT cell responses were recorded (experimental flow sessions only). The majority of cumulative response power for each glomerulus, 88.5 ± 7.9% (mean ± st. dev), was also found to be within this range. Thus, the majority of response power for each glomerulus was measured to be within a relative frequency range of the stimulus (Figures 7A,B). Across all glomeruli recorded, response power was not significantly different between flow conditions. To examine the effect of flow conditions on the response power of cells that most strongly responded to the odor, we next analyzed only glomeruli whose mean response was above the 75th percentile. Within this group of glomeruli, response power did change significantly between flow [*t*_(27)_ = 5.52, *p* < 0.001], with stronger response power during high flow conditions (Figures 7D,E). This increase reflects the significant increase in stimulus power (0–5 Hz) observed in high flow as compared to low flow trials [*t*_(116)_ = 31, *p* < 0.001] (Figure 7C). Thus, the response power of glomeruli with the strongest signals was significantly affected by flow condition.

There exists a relationship between each of these two response features, responsivity and response power, and how well a glomerulus follows plume dynamics. Across all trials, glomeruli with higher responsivity to plume presentations were better at following changes in odor concentration (*r* = 0.76, *p* < 0.001). Thus, the more reliably a glomerulus responded to plume presentations, the more likely it was to better follow changes in odor concentration (Figure 6C). These findings were reflected in the supplementary odor panel study, where glomeruli with higher responsivity to plumes had responses that were more correlated with plume dynamics [*F*_(2,116)_ = 8.06, *p* < 0.001] (Supplementary Figure 8E sorted bottom to top by increasing correlation magnitude). This is not a perfect relationship as glomeruli that are responsive to the plume but not its dynamics exist (Figure 6A), but a glomerulus with higher responsivity is more likely to be correlated to plume dynamics than one with lower responsivity. As mentioned previously, a glomerulus's average responsivity level is also moderated by flow condition. Thus, a glomerulus's responsivity predicts its ability to track plume dynamics and is moderated by changes in dynamic regimes.

As for the second response feature, response power was also correlated with how well the glomerulus followed changes in odor concentration, when averaged across all trials glomeruli with higher response power were significantly better at following plume dynamics (*r* = 0.74, *p* < 0.001) (Figure 7E). When this relationship was examined within flow conditions (Figure 7C), high flow was significantly correlated (*r* = 0.73, *p* < 0.001), but low flow was no longer significantly correlated (*r* = 0.19, *p* = 0.05). This significant correlation in high flow was driven by a subset of the strongest responding glomeruli (75th percentile) whose response power was significantly moderated by flow condition. This relationship between response power and tracking ability (correlation with plume dynamics) was also observed in the supplementary odor panel study where higher average response power predicted higher correlations between that response and plume dynamics [*F*_(2,116)_ = 29.2, *p* < 0.001] (Supplementary Figure 8C). Thus, a glomerulus's response power predicts its ability to track plume dynamics and for stronger responders, this relationship is moderated by changes in dynamic regimes.

These results suggest that both the reliability and the temporal pattern of MT activity is significantly moderated by the odor concentration dynamics of the incoming stimuli. Thus, the spatiotemporal dynamics of plumes play a role in structuring activity in the first olfactory relay of the mouse's brain during natural olfactory processing.

## 4. Discussion

Mice are adept at olfactory guided search despite the stochasticity and complexity of odor plumes used in navigation. Spatiotemporal cues present in natural odor scenes are thought to drive decision-making in olfactory search (Mafra-Neto and Cardé, 1994; Vickers, 2006; Pang et al., 2018), but how they moderate population activity in the olfactory bulb (OB) is unknown. Releasing odor within a custom-built wind tunnel, we were able to hold constant all properties of the odor stimulus and the animal's position relative to the source and vary only the air velocity through which the plume traveled. By using this approach we altered the Reynolds number of the flow and created plumes with varying statistical structures and odor concentration dynamics. In this way, the effect of plume dynamics on MT population activity could be examined using naturally evolving odor plumes. Recording MT activity in mice expressing GCaMP6f, we show that a significant fraction of glomerular populations of MT cells follow odor plume dynamics. Additionally, the strength with which they do so is moderated by airflow, such that increased flow velocity and turbulence (Reynolds number) results in increased correlation of MT cell activity with plume dynamics. This work shows that plume dynamics structure the activity of the OB, the first relay of olfactory coding in the mouse's brain.

The recent history of an odor stimulus has been shown to be present in olfactory encoding in both serial sampling of odor concentration in mice (Parabucki et al., 2019) and tracking of odor concentration in invertebrates (Geffen et al., 2009), showing odor concentration changes influence olfactory encoding. Although inter-sniff comparisons in mice show that MT cells can detect the sign and magnitude of changes in odor concentration (Parabucki et al., 2019), it is unknown whether they are able to resolve the dynamics of natural plumes, which span across a range of temporal scales. If odor concentration dynamics are resolved, computational work has shown that they are informative for olfactory search (Baker et al., 2018; Gumaste et al., 2020). To avoid the complexity of stochastic odor plumes, the averaging of odor concentration dynamics could be an alternative strategy to navigate olfactory environments. Mean odor concentration levels are moderated both by the distance from an odor source and by how close an animal is to the central stream of the plume (Crimaldi and Koseff, 2001). While this measure is potentially informative, it does not by itself sufficiently inform decision-making on the timescale observed in rodents (Gumaste et al., 2020). Therefore, it is likely that the mouse relies upon spatiotemporal features of the plume for olfactory search as information can be extracted from odor concentration dynamics (Baker et al., 2018).

Our study found a correlation between MT activity and odor concentration dynamics during plume presentations. The temporal information conveyed by MT cells could support a variety of navigation algorithms. For instance, two important dynamic features are the length of odor encounters, whiffs, and the timing between odor encounters, blanks. Whiff and blank duration are moderated by the distance between an animal and the odor source. As an animal approaches an odor source, plume encounters become shorter and more frequent (Wright and Thomson, 2005; Celani et al., 2014). Blank duration has been shown to be particularly informative even when olfactory environments change. Computational modeling of olfactory search in invertebrates (Park et al., 2016; Rapp and Nawrot, 2020) as well as fluid dynamics modeling (Celani et al., 2014) shows that the time between odor encounters, blank duration, is less sensitive to environmental conditions, such as plume velocity or potency of the odor source that are known to affect interpretation of odor concentration dynamics (Webster and Weissburg, 2001; Connor et al., 2018). Specifically, Park et al., found blank duration to be a more efficient source of information for olfactory search than instantaneous tracking of odor concentration. In our study, we observed that MT activity was more correlated with plume dynamics in high flow trials than low flow trials. Odor concentration in low flow trials was less skewed, meaning that these trials had lower intermittency and odor concentration tended to fluctuate around a central value. Alternatively, high flow trials were more skewed and were characterized by a whiff and blank structure. The fact that correlations are higher in high flow suggests MT activity may be more responsive to whiff and blank features as opposed to tracking fine fluctuations in odor concentration across more constant plume encounters. One limitation of this study is that medium flow was not statistically distinguishable from high flow, therefore, we were unable to include intermediary intermittency levels between low and high flow conditions in our analysis. Future studies exploring the effect of a broader range of intermittency levels on MT activity during plume encounters could help determine which spatiotemporal features of intermittency are moderating MT responses to plume dynamics.

A network where the majority of glomerular activity responds to concentration dynamics could be considered to be inefficient when the OB has to perform other tasks, such as odor identification and segmentation. Glomerular spatial maps, i.e., glomerular ensembles consistently responding to an odor, are thought to be one of the primary means of odor identification (Wachowiak and Shipley, 2006). Odors maps vary with concentration (Xu et al., 2000; Wachowiak and Cohen, 2001), but are stable enough to reliably encode odor identity (Belluscio and Katz, 2001). Although pulsed odors can be rapidly discriminated (<200 ms, or a single sniff, Uchida and Mainen, 2003), in a natural olfactory environment where odors are intermittent (Murlis et al., 2000; Celani et al., 2014) and mixed, identification becomes a much more complicated task especially for identification of mixtures. Glomerular ensembles reliably responding across odor encounters could aid odor discrimination in mixed odor environments. Spatial maps of odor identification will overlap in natural olfactory scenes where an animal encounters signals from multiple odor sources as it navigates a plume. Odors co-released travel together (Celani et al., 2014), and therefore a mixture of odors emanating from the same source will have correlated temporal dynamics in the plume and will thus be experienced by a searcher as having correlated encounters across whiffs. This means the probability of the signal from separate sources arriving together reliably across whiffs would be low if odors are released from spatially separated sources. Grouping and demixing these odor representations using the correlation, or lack thereof, in the odor concentration dynamics could aid odor discrimination in complex environments (Hopfield, 1991).

Since our studies are not recorded at the individual cell level, the potential degree of heterogeneous tuning to different features among MT cells within a single glomerulus was not examined. It could be that observed correlations of glomerular MT populations were a product of the collective activity of heterogeneously tuned MT cells within a glomerulus, but MT responses have been shown to linearly sum odor inputs (Gupta et al., 2015), which contradict the idea that they are directly tuned to different features of plume dynamics. At the same time, this does not infer MT activity responding to plume dynamics is homogeneous as the responsiveness (Adam et al., 2014) and the response (Geramita and Urban, 2017) varies between MT cells across concentration levels (Cleland and Borthakur, 2020). Future research across a variety of odor concentration dynamic regimes and odor mixtures at both the cellular and population level are needed to further investigate the degree to which bulbar responses are tuned to features of odor concentration dynamics and how this tuning may impact optimal encoding of odor information.

Sniff frequencies are known to influence bulbar oscillations, and thus if sniffing behavior varied significantly between flow conditions, this may have contributed to some of the observed differences in tracking behavior between low and high flow trials. For example, it could be that faster stimulus dynamics in high flow may cause the mouse to miss features of the dynamics due to an inability to resolve them with sufficient sampling speed. However, recent work suggests sniff frequency does not necessarily limit the resolution of plume frequencies in such a manner (Dasgupta et al., 2020). Dasgupta et al. (2020) found subthreshold MT activity was able to couple with frequencies of odor pulsed at supra-sniff frequencies. Thus, if sniff frequency was moderated significantly between flow conditions, it would not necessarily set an absolute bound on the resolution of plume dynamics. We believe it is important to monitor sniff frequency in the future to observe how it affects inter-sniff and intra-sniff activity, and consequently how these changes relate to tracking behavior observed in MT populations across flow conditions.

Our data show that MT activity in the OB of mice follows the temporal dynamics of odor plumes. Additionally, we demonstrate that this effect is stronger under conditions that generate larger Reynolds numbers. Following odor concentration dynamics within plumes could enable MT cells to convey information useful for olfactory search. Following the temporal dynamics of odor plumes may also be an efficient form of multiplexing odor identity and source location for the first olfactory relay in mice. Although MT activity responds to changes in odor concentration, the observed correlations do not suggest perfect tracking at the level of individual glomeruli and indicate inter-individual differences in the degree to which glomeruli follow plume dynamics. Future research focusing on location encoding across a wide range of both intermittency regimes and odor panels is needed to clarify the degree to which bulbar activity is tuned to features of plume dynamics and how a balance between identity coding and concentration coding is instrumental in supporting the wide variety of behaviors enabled by olfaction.

## Data Availability Statement

The data supporting the conclusions of this article will be made available by the authors, without undue reservation.

## Ethics Statement

The animal study was reviewed and approved by Institutional Animal Care and Use Committee (IACUC) of the University of Washington.

## Author Contributions

DG, AS, and SL initiated the study and designed the experiments. DG and SL built the head-fixed imaging setup, built the plume delivery setup, and wrote the acquisition software. SL and LX performed the cranial window surgeries. SL, LX, and LS performed the experiments. DG, MS, and SL performed the analysis of neural data. DG, AS, SL, and NR performed the analysis of plume dynamics, developed the deconvolution based on Tariq et al. (2021) (MT) and MT assisted with sensor characterization. DG, AS, SL, and NR generated the figures reporting data with input from MS. All authors helped with the text.

## Conflict of Interest

The authors declare that the research was conducted in the absence of any commercial or financial relationships that could be construed as a potential conflict of interest.
