# Supplementary material for: Plume Dynamics Structure the Spatiotemporal Activity of Mitral/Tufted Cell Networks in the Mouse Olfactory Bulb
Source: Front Cell Neurosci. 2021 Apr 30;15:633757. doi: 10.3389/fncel.2021.633757 (PMC8127944; doi:10.3389/fncel.2021.633757)
Supplement: Supplementary file 1 [file Data_Sheet_1.pdf]

Supplementary Material

1 SUPPLEMENTARY DATA

2 SUPPLEMENTARY TABLES AND FIGURES

2.1 Figures

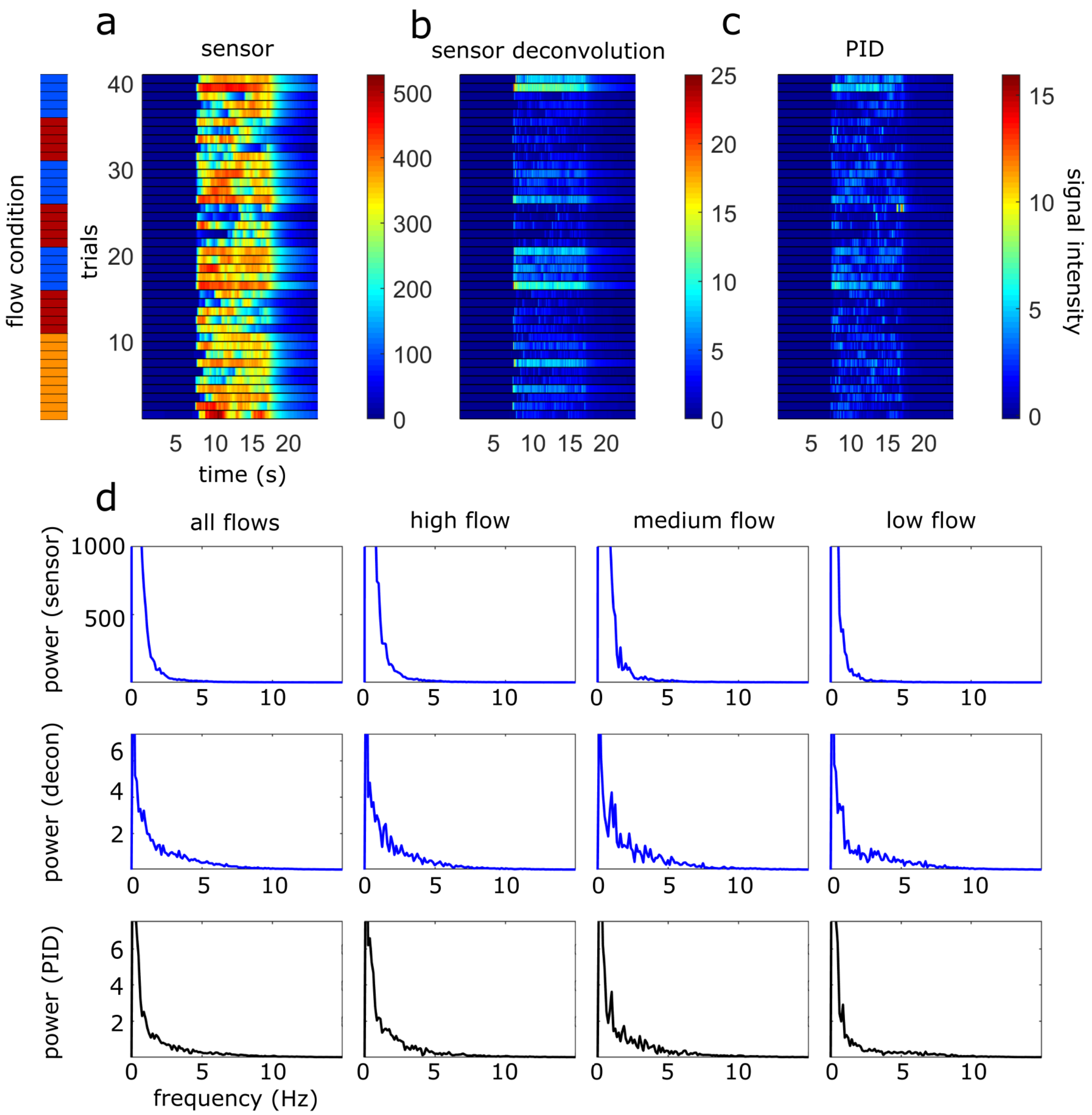

Figure S1: **Deconvolved Ethanol signal and spectral decomposition from one session divided by flow type.** a) The raw ethanol sensor output is shown for all trials within a single session (n=40). (red, orange, and blue flow condition depict high, medium and low flow respectively). b) The deconvolution of the raw ethanol signal. c) The simultaneously recorded signal from a co-localized PID. Sensor deconvolution and PID are significantly correlated as calculated during plume presentations ($r = .3089$, $p < .001$). d) (top) The spectral decomposition calculated across all trials or within flow conditions for the raw sensor signal shown in (a). Only the middle 8 seconds of the 10 second plume presentation are analyzed to avoid onset or offset dynamics. Spectral decompositions are also plotted for the ethanol deconvolution (middle) shown in (b) and for the PID (bottom) shown in (c).

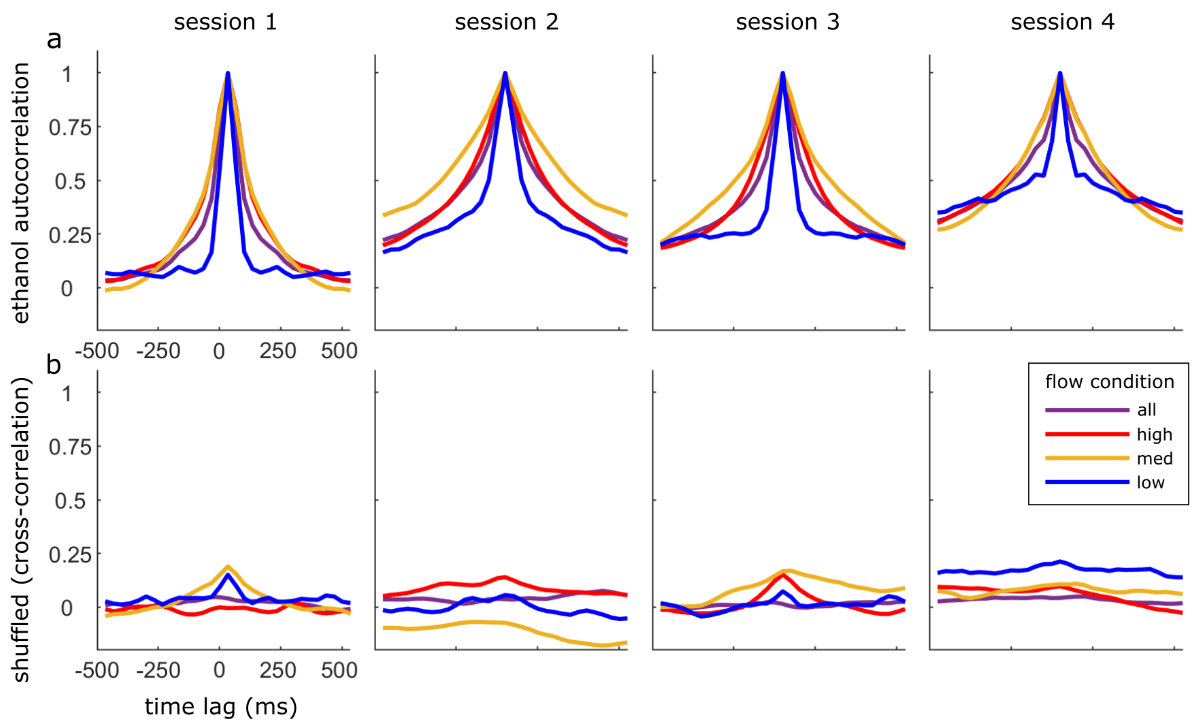

Figure S2: **Normalized Autocorrelation of Ethanol trace.** a) The Normalized average autocorrelation of the deconvolved ethanol signal is plotted for each of the 4 experimental sessions showing that for all flow conditions, plume dynamics are stochastic and do not correlate across time within trials. Traces are calculated across all trials within the designated flow conditions. b) Same as (a) but trials are shuffled within session such that each cross-correlation is calculated between the deconvolved ethanol signal from two different trials. Plotted cross-correlations show concentration dynamics are not correlated across plumes.

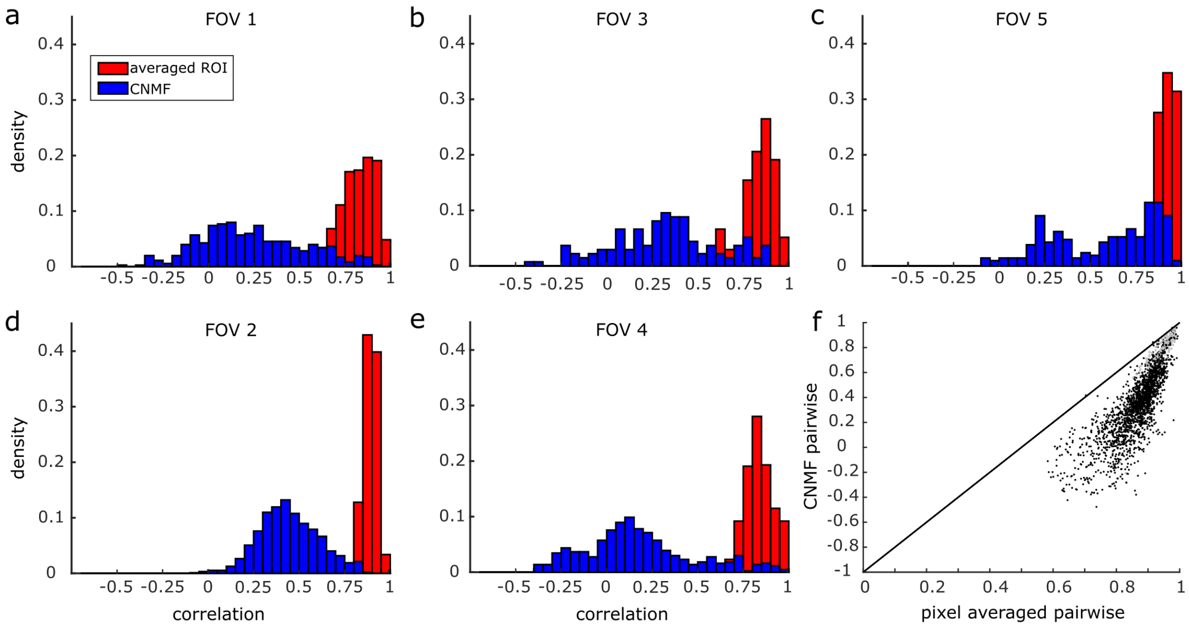

Figure S3: **Pairwise correlations between glomerular ROIs are decorrelated by CNMF.** a-e) Distributions of the pairwise linear correlation coefficients from all possible glomerular pairs within each FOV. Correlations are calculated on the temporal decomposition of CNMF ROI signals (without deconvolution) which include denoising and demixing (blue) or on the raw pixel values as averaged within the same ROI boundaries (red). Pairwise correlations are calculated between the two glomeruli during stimulus presentation. f) Scatterplot of each pairwise correlation shows CNMF denoising decorrelates the signal for each pair of glomeruli. Pairwise correlations in grey are from ROI pairs that have been considered to originate from the same glomerulus after the merging threshold analysis (Supplemental Fig. 4).

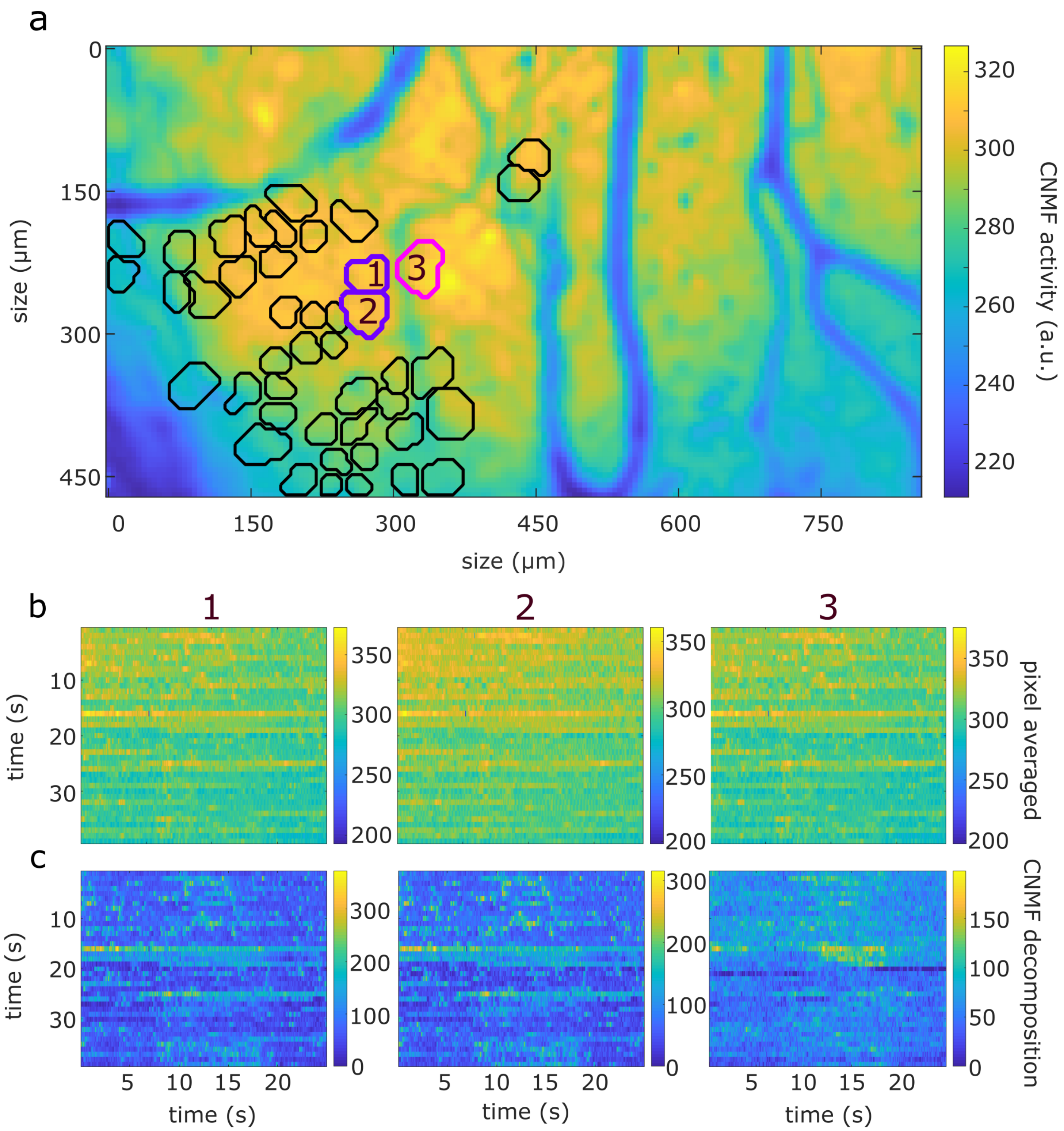

Figure S4: **Detecting oversegmentation of ROIs after CNMF decomposition.** a) ROI segmentation of glomeruli for FOV 4 using CNMF spatial decomposition of activity across an entire session. ROIs circled in purple do not meet thresholds for independent ROIs (methods) and thus are considered to originate from the same glomerulus, while the ROI in magenta is considered to be a separate glomerulus. b) Raw fluorescent signal averaged across all pixels within each of the 3 ROIs (without denoising or de-mixing) is plotted for all trials in the session. Numbers above each plot refer to ROI numbering in panel (a). c) CNMF signal (without deconvolution) across all trials plotted for the corresponding ROIs in (b).

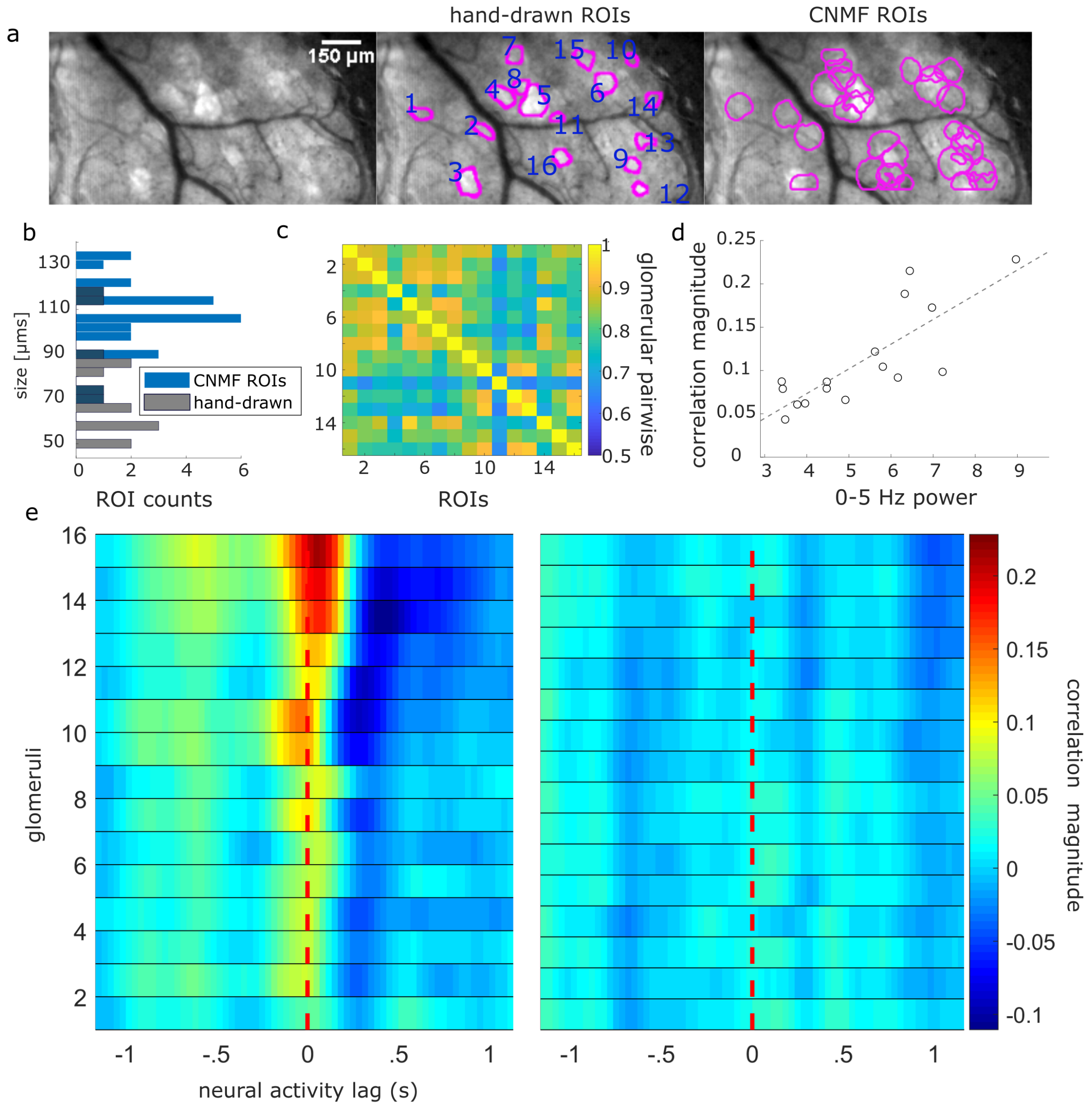

Figure S5: **Hand-drawn ROIs have qualitatively similar results but higher pairwise correlations.** a) (left) St. dev projection of a single FOV during the first trial of the session. (middle) ROIs from hand-drawn analysis are plotted over the projected st. dev (left). ROIs determined using recordings and st. dev projections averaged across trials within each flow condition. (right) Same but CNMF ROIs are plotted. b) Distribution of hand-drawn ROI sizes (grey) plotted against the CNMF ROI sizes (blue) found for the same FOV. The size distribution of the hand-drawn analysis was significantly smaller than that of the CNMF analysis ($z = 3.94$, $p < 0.001$). c) Trial averaged linear pairwise correlation coefficients of ROI activity during plume presentations shows high correlations between ROIs. d) A glomerulus's correlation with odor dynamics (tracking/correlation magnitude) is plotted against their corresponding change in response power ($0 - 5$Hz) between 'odor off' and 'odor on' periods showing glomeruli with higher correlation coefficients have higher activity power ($r = 0.80$, $p =< 0.001$). The dotted line plots the line of best fit using OLS regression. f) Glomerular activity is correlated with stimulus dynamics at varying lags (left) with all mean coefficients exceeding a 95% confidence interval of their shuffled mean coefficients. Each row is a glomerulus (hand-drawn ROI) and each time point represents the mean correlation coefficient between odor and response deconvolutions at the indicated lag. Glomeruli are sorted in order of decreasing correlation. (right) Same but neural activity responses are shuffled so that the signals compared are not from the same trial. The glomeruli are sorted to match their corresponding unshuffled cross-correlation in the left panel.

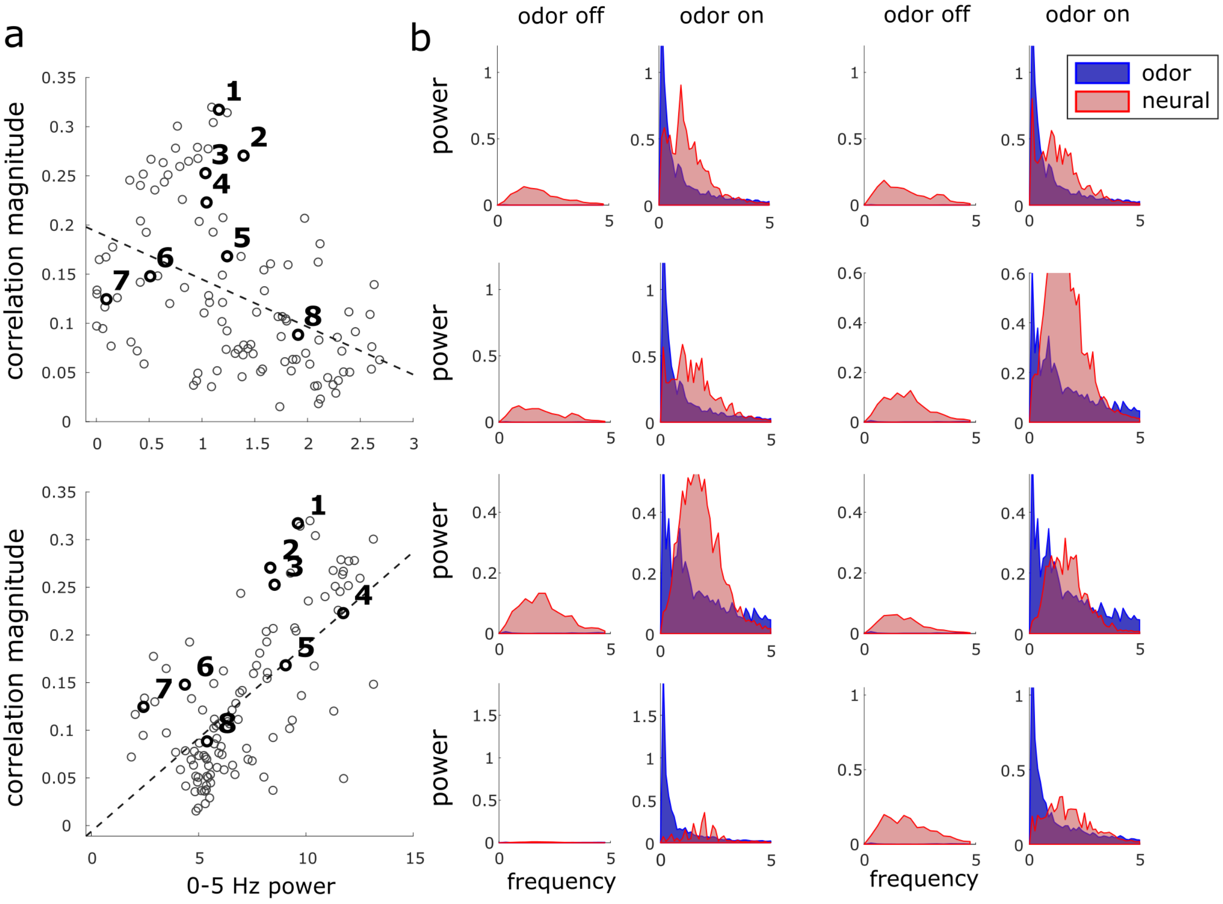

Figure S6: **0-5 Hz Power spectrums for odor and glomerular responses.** Average correlation magnitude (tracking ability) for each glomerulus is plotted against the power spectrum of its activity during ‘odor off’ (top) or ‘odor on’ (bottom) periods, illustrating the before and after states of the change in response power plotted in Figure 6d. b) The average power spectrums across trials of the glomerular (red) traces and the deconvolved odor (blue) to which the glomerulus responses are plotted for 8 example glomeruli (numbered accordingly in (a)) during ‘odor off’ and ‘odor on’ periods.

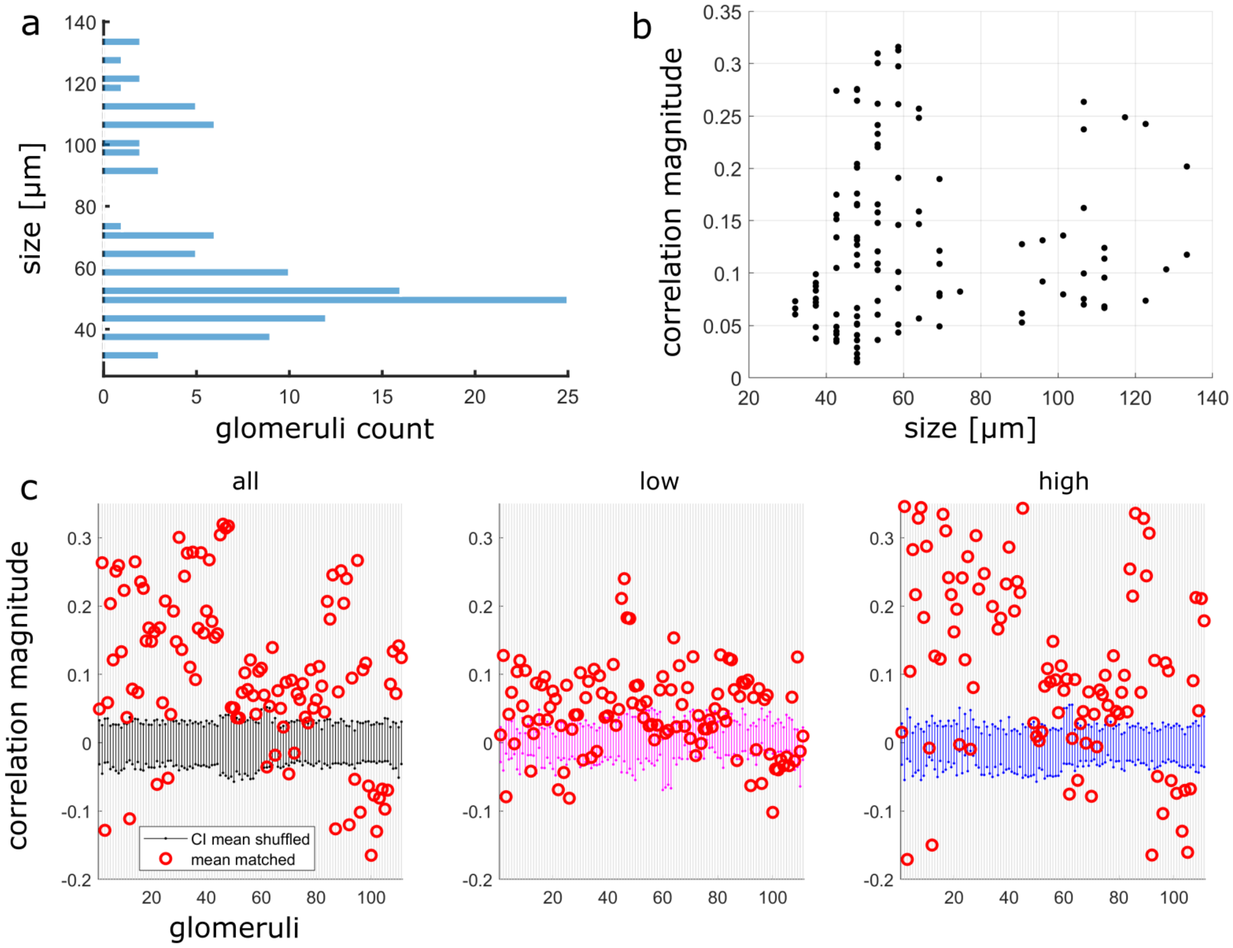

Figure S7: **Glomerular sizing and plume following behavior.** a) Distribution of CNMF ROI sizes for all sessions (μm). b) ROI size (μm) plotted against the mean correlation coefficient between deconvolved glomerular and ethanol signals during odor presentation shows correlations between ROIs and plume dynamics are not driven exclusively by singular MT cell activity. Kendall's tau coefficient between size and odor concentration tracking ($r_\tau = 0.1563$, $p = 0.202$) shows that correlation to plume dynamics is not exclusive to or related to smaller sized ROIs (ROIs similarly sized to individual MT cells). This suggests response to plume dynamics across plume encounters is also occurring at the glomerular level. c) Mean correlation coefficients of glomeruli (red) are plotted against their respective bootstrapped 95% confidence interval of null mean coefficients (see methods for trial shuffled bootstrap analysis). Null confidence intervals are calculated across all flow conditions (black), within low (pink) and within high (blue) flow.

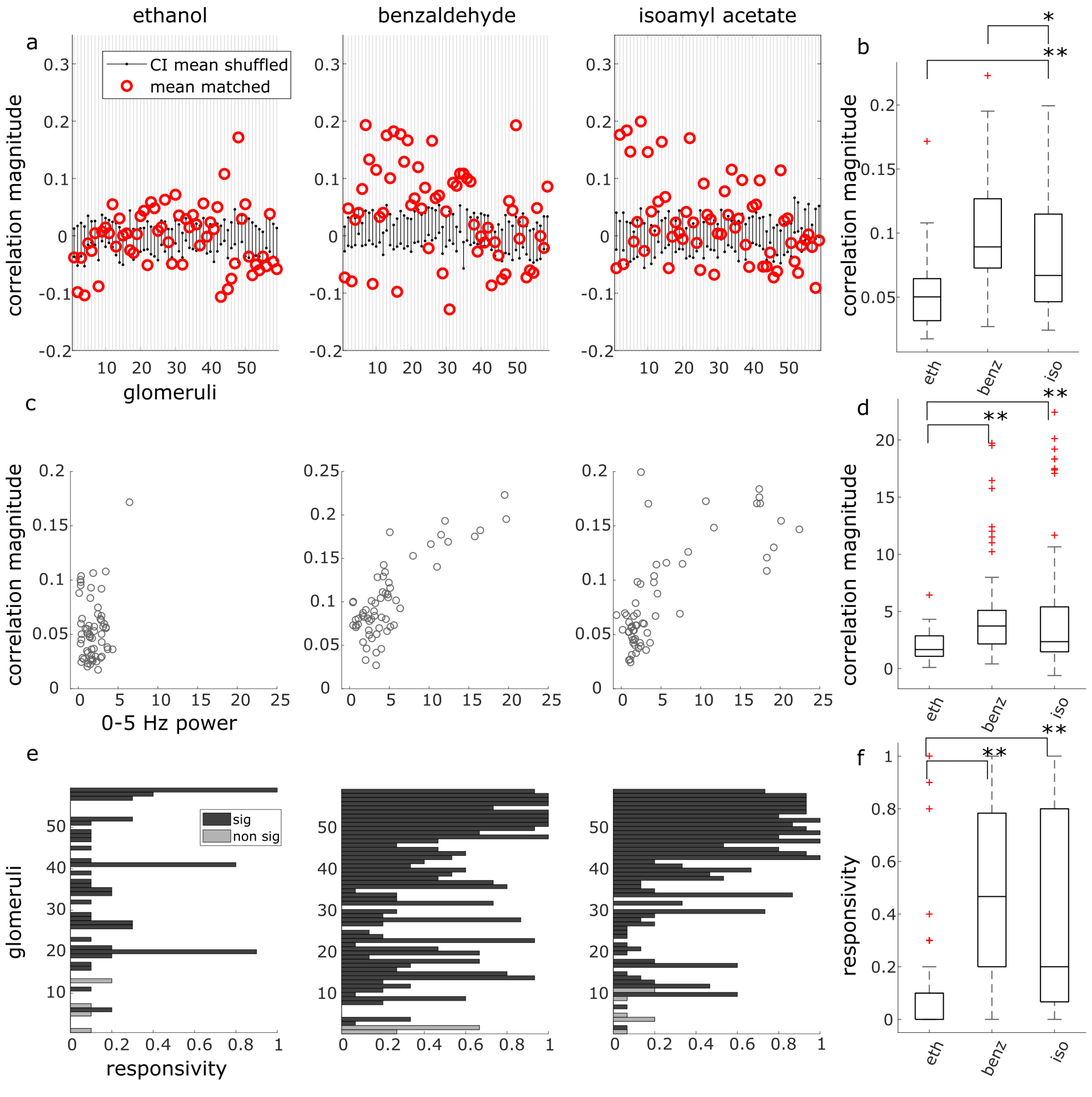

Figure S8: **Significant tracking of odor concentration dynamics is observed across odors.** a) Mean correlation coefficients of glomeruli (red) are plotted against their respective bootstrapped 95% confidence interval of null mean coefficients (black). Null confidence intervals are calculated within odor type. b) Box plot shows absolute value distributions of correlation magnitude plotted for each odor in (a) where the central line indicates the median, boxes indicate 25th and 75th percentiles, whiskers indicate min and max distribution values, and outliers are plotted in red. c) Calculated within odor type, correlation magnitude (tracking ability) of the glomerulus with plume dynamics is plotted against its corresponding increase in response power (0-5Hz) activity between 'odor off' and 'odor on' periods. This relationship is significantly correlated for both odor mixtures, but not for ethanol alone(r = 0.12, p = 0.152, r = 0.81, p = ¡0.001, r = 0.74; p = ¡0.001; ethanol, benzaldehyde, and isoamyl acetate respectively). d) Box plot of change in response power distributions for the glomerular population for each odor depicted in (c). Repeated measures ANOVA shows significant differences in response power change of the glomerular population between ethanol and odor mixtures ($F(2, 116) = 8.06, p < 0.001$). e) The responsivity of each glomerulus to the specified odor is plotted. Glomeruli are sorted by decreasing average correlation with plume dynamics during the specified odor (top to bottom). Glomeruli whose correlation coefficient exceeds

Figure S8 *(previous page)*: its null confidence interval in at least one odor condition are plotted in dark grey as significantly tracking (sig, n=54) and the minority of glomeruli that do not (non sig, n=5) are plotted in light grey. Responsivity was significantly correlated with tracking ability for all odor conditions ($r = 0.36$, $p < 0.01$, $r = 0.66$, $p =< 0.001$, $r = 0.82$, $p < 0.001$; ethanol, benzaldehyde, and isoamyl acetate respectively). f) Box plot of responsivity distributions for the glomerular population for each odor depicted in (f). Repeated measures ANOVA shows significant differences in responsivity between ethanol and odor mixtures ($F(2, 116) = 29.2$, $p < 0.001$).
